# Cell Cycle-Dependent Phosphorylation of *Theileria annulata* Schizont Surface Proteins

**DOI:** 10.1371/journal.pone.0103821

**Published:** 2014-07-31

**Authors:** Olga Wiens, Dong Xia, Conrad von Schubert, Jonathan M. Wastling, Dirk A. E. Dobbelaere, Volker T. Heussler, Kerry L. Woods

**Affiliations:** 1 Division of Molecular Pathobiology, Vetsuisse Faculty, University of Bern, Bern, Switzerland; 2 Department of Infection Biology, Institute of Infection and Global Health & School of Veterinary Science, University of Liverpool, Liverpool, England; 3 The National Institute for Health Research Health Protection Research Unit in Emerging and Zoonotic Infections, University of Liverpool, Liverpool, England; 4 Institute of Cell Biology, University of Bern, Bern, Switzerland; University of Padova, Italy

## Abstract

The invasion of *Theileria* sporozoites into bovine leukocytes is rapidly followed by the destruction of the surrounding host cell membrane, allowing the parasite to establish its niche within the host cell cytoplasm. *Theileria* infection induces host cell transformation, characterised by increased host cell proliferation and invasiveness, and the activation of anti-apoptotic genes. This process is strictly dependent on the presence of a viable parasite. Several host cell kinases, including PI3-K, JNK, CK2 and Src-family kinases, are constitutively activated in *Theileria*-infected cells and contribute to the transformed phenotype. Although a number of host cell molecules, including IkB kinase and polo-like kinase 1 (Plk1), are recruited to the schizont surface, very little is known about the schizont molecules involved in host-parasite interactions. In this study we used immunofluorescence to detect phosphorylated threonine (p-Thr), serine (p-Ser) and threonine-proline (p-Thr-Pro) epitopes on the schizont during host cell cycle progression, revealing extensive schizont phosphorylation during host cell interphase. Furthermore, we established a quick protocol to isolate schizonts from infected macrophages following synchronisation in S-phase or mitosis, and used mass spectrometry to detect phosphorylated schizont proteins. In total, 65 phosphorylated *Theileria* proteins were detected, 15 of which are potentially secreted or expressed on the surface of the schizont and thus may be targets for host cell kinases. In particular, we describe the cell cycle-dependent phosphorylation of two *T. annulata* surface proteins, TaSP and p104, both of which are highly phosphorylated during host cell S-phase. TaSP and p104 are involved in mediating interactions between the parasite and the host cell cytoskeleton, which is crucial for the persistence of the parasite within the dividing host cell and the maintenance of the transformed state.

## Introduction

The transforming parasites *Theileria annulata* and *T. parva* belong to the Apicomplexan phylum that also includes *Toxoplasma* and *Plasmodium* spp. *T. annulata* and *T. parva* invade bovine leukocytes and are the causative agents of the leukaemia-like diseases Tropical Theileriosis and East Cost Fever (ECF), respectively. In contrast to *Plasmodium* and *Toxoplasma*, *Theileria* rapidly destroys the surrounding host cell membrane following invasion and associates with host cell microtubules, thus establishing its niche in the leukocyte cytoplasm [1]. Once free in the cytoplasm the *Theileria* sporozoite differentiates into a multi-nucleated schizont which, uniquely for a eukaryotic cell, reversibly transforms the host cell (reviewed in [2]).

*Theileria*-dependent transformation results in the uncontrolled proliferation of the infected cell driven by autocrine factors [3]–[5]. Parasitised cells become resistant to apoptosis [6] and acquire increased invasiveness and a metastasic phenotype [7]–[9]. Importantly, the transformed phenotype of infected cells is entirely reversible upon killing the parasite, making *Theileria*-induced transformation a unique model to study leukocyte transformation. Several host cell kinases including phosphoinositide 3-kinase (PI3-K,) Src family kinases, casein kinase II (CK2), protein kinase A (PKA), Akt/PKB, and c-Jun N-terminal kinase (JNK), and downstream transcription factors, are constitutively activated in *Theileria*-infected cells in a parasite-dependent manner and contribute to the transformed phenotype [4], [10]–[20]. While the modification of host cell signalling pathways in response to *Theileria* infection has been quite thoroughly studied, very little is known about the parasite factors involved.

Recently the first analysis of the *Theileria* proteome was published, in which 21.5% (812 proteins) of all predicted *T. annulata* schizont proteins were detected in lysates from purified parasites and following parasite membrane enrichment [21]. Schizont proteins that are predicted to be expressed on the parasite surface or secreted into the cytoplasm are of particular interest as potential modifiers of host phenotype, and in this context it is rather surprising that no obvious *Theileria*-encoded kinases or phosphatases possess a predicted signal peptide or transmembrane domain(s) [22], [23].

The cytoplasm-dwelling schizont is strictly intracellular, and ensures its persistence within continuously proliferating host cells by associating with the mitotic apparatus and by becoming “incorporated” into the central spindle during host cell cytokinesis [24]. As part of this process, the host cell mitotic kinase polo-like kinase 1 (Plk1) is recruited to the schizont surface in a cell-cycle dependent manner, and its kinase activity was found to be essential for the association of the parasite with central spindles. Binding of Plk1 to the schizont surface is negatively regulated by the activity of host cell CDK1, but the parasite ligand(s) involved in the interaction, and the identity of parasite-associated Plk1 substrate(s), remain unknown. More recently we reported that EB1, an important regulator of microtubule dynamics, also binds to the parasite surface in a cell cycle-dependent manner via a specific EB1-binding motif present in the *Theileria* surface protein p104 [25]. Plk1 is not the only host cell kinase found to associate with the schizont membrane. One central feature of *Theileria*-induced transformation is the parasite-dependent constitutive activation of the transcription factor NF-kB [26], and this is mediated by the formation of activated IkB kinase (IKK) signalosomes at the parasite surface [18]. Again, the parasite ligand(s) involved in this interaction are unknown.

Considering the extensive changes in kinase activity that occur in response to *Theileria* infection, and also the cell cycle-dependent regulation of Plk1 and EB1 association with the schizont, we became interested in analysing phosphorylation events that occur at the parasite surface. We made use of antibodies that specifically detect phospho-threonine (p-Thr), phospho-threonine-proline (p-Thr-Pro) and phospho-serine (p-Ser) epitopes, and observed significant phosphorylation of the schizont during host cell interphase. The availability of well-established protocols to synchronise parasitised cells in specific phases of the cell cycle [24], [27] prompted us to perform label-free mass spectrometry analysis on schizonts purified from cells blocked in S-phase and mitosis. We identified 65 phosphorylated schizont proteins, including 15 that possess a predicted signal peptide and/or transmembrane domain, and thus have the potential to be targeted by host cell kinases. In particular we describe cell cycle-specific phosphorylation of two important surface antigens, p104 (TA08425) and TaSP (TA17315), which are involved in interactions between the parasite and the host cell cytoskeleton [25], [28].

## Materials and Methods

### Cell culture, flow cytometry & parasite enrichment

TaC12 is a *T. annulata* schizont-infected cell line obtained by *in vitro* infection of peripheral blood cells [29]. BoMAC is an SV40-transformed cell line of *Theileria*-uninfected bovine macrophages [30]. Both cell lines were cultured as described previously [24]. For cell cycle arrest in prometaphase, cells were treated with 0.1 µg/ml nocodazole (Biotrend) for 16 h, and harvested by shake-off. For synchronisation in S-phase cells were incubated for 24 hours in medium containing 4 mM thymidine, as described [24]. For flow cytometry analysis, TaC12 cells were washed with PBS, and analysed as described [31]. Raw data analysis was performed using the cytometric analytic software Flow JoX. For parasite enrichment 10^8^ TaC12 cells (per sample) were incubated for 1 hour in medium containing 3 µg/ml nocodazole to depolymerise microtubules, then washed once in ice cold PBS (5 min at 200×g). The host cell membrane was perforated using activated aerolysin (Peter Howard, Department of Microbiology and Immunology; University of Saskatchewan; Saskatoon Sasktachewan Canada) essentially as described [27]. Briefly, cells were resuspended in ice-cold HEPES buffer (10 mM HEPES, 150 mM NaCl, 20 mM KCl, pH 7.4) containing 1 mM CaCl_2_ and incubated with 50 µg aerolysin for 1 hour on ice. This was carried out in the presence of 50 nM calyculin A (Millipore) to minimise dephosphorylation. Unbound aerolysin was washed away with 10 ml HEPES buffer containing 1 mM CaCl_2_, and cell pellets were resuspended in an equal volume of HEPES buffer containing 1 mM CaCl_2_ and 50 nM calyculin A. Mitotic samples were additionally treated with 2 µl/ml DNase (Benzonase Nuclease, >250 units/µl, Sigma). Cells were incubated at 37°C for 30 min to allow host cell membrane perforation, and subjected to mechanical lysis using a syringe (TERUMO, NN-2070S; 20 Gx2 ¾; 0.9×70 mm). A 50% Nycodenz (Axon) stock-solution (w/v) was prepared in buffered solution (0.128 M NaCl, 5 mM Tris-HCl (pH 7.5) containing 3 mM KCl and 0.3 mM EGTA), and used to make 40%, 30% and 5% Nycodenz solutions in 1× PBS. A Nycodenz step gradient was prepared in 30 ml COREX tubes (No 8445) consisting of 15 ml 5% Nycodenz solution underlaid with 5 ml 30% and 1.5 ml 40%. 1 ml cell suspension was loaded on top of the gradient and centrifuged at 450×g at 18°C for 20 min. The fraction between the 5% and 30% phases was collected and washed in 50 ml PBS, pelleted (10 min at 450×g), snap-frozen in liquid nitrogen and stored at −80°C.

### Generation of a rat polyclonal anti-*Theileria* schizont antibody

Schizonts were purified from unsynchronised TaC12 cells. One rat was immunised three times with 60 µg schizont protein suspension (per injection) resuspended 1∶1 in PBS and GERBU Adjuvant 100 (3100). This work was carried out at the central animal facility of the University of Bern in strict accordance to the guidelines of the Swiss Tierschutzgesetz (TSchG; Animal Rights Laws) and European regulations, and approved by the “Amt für Landwirtschaft und Natur” in Bern (Permit Number: BE105/10).

### Immunofluorescence microscopy & Western blotting

The following primary antibodies were used: mouse mAb 1C12 (anti-p104) and the rabbit polyclonal anti-TaSP were used as described [25]. Anti-α-tubulin (clone DM1A, Sigma, 1∶3000 dilution), rat polyclonal anti-*T. annulata* schizont antibody (1∶1000), mouse mAb anti-*T. parva* HSP70 [32] 1∶2000 dilution, mouse mAb anti-p-Thr-Pro (Cell signalling; 9391, 1∶1000 dilution), mouse anti-p-Ser (BD Transduction Laboratories TM, 1∶3000), rabbit polyclonal antibody anti-p-Thr (Cell signalling; 9381, 1∶3000), mouse mAb p-Tyr-100 (Cell signalling, 9411 1:1000). Mouse anti-BrdU (Clone G3G4; mouse IgG1, kappa light chain, University of Illinois). For IFA secondary antibodies conjugated with Alexa Fluor 488 or Texas Red (Molecular Probes) were used. Cells were fixed and permeabilised for microscopy using 4% PFA or ice-cold methanol as described [25]. For analysis of host and parasite DNA synthesis, TaC12 cells were synchronised in S-phase as described above, and incubated with 10 µM BrdU for 2 h at 37°C prior to fixation with 4% PFA and analysis with anti-BrdU antibodies. DNA was labelled using DAPI and cells were mounted using DAKO mounting media. Wide-field fluorescence microscopy was performed with a Nikon Eclipse 80i microscope as described [25], and images processed using Photoshop. For Western blotting cell pellets (TaC12 cells and purified schizonts) were lysed for 30 minutes in 8 M urea lysis buffer (8 M urea (freshly prepared), 100 mM ammonium carbonate pH 8, 1× protease inhibitor mix (Roche), 100 nM calyculin A), briefly sonicated, and the lysate supernatant obtained by centrifuging for 5 min at 16,000×g.

### LC-MS/MS analysis

Protein from *T. annulata* schizont samples was dispensed into low protein-binding microcentrifuge tubes (Sarstedt, Leicester, UK) and made up to 160 µl by addition of 25 mM ammonium bicarbonate, 10 mM NaF, 300 µM Na_3_VO_4_, 1 mM benzamidine, 2 µM PMSF, 10 mM beta-glycerophosphate and 1× Sigma Phosphatase inhibitor cocktail 2 (Sigma-Aldrich), 1× mini EDTA free protease inhibitor cocktail (Roche). Proteins were denatured using 100 µl of 1% (w/v) RapiGest™ (Waters MS Technologies, Manchester, UK) in 25 mM ammonium bicarbonate followed by three cycles of freeze-thaw, and two cycles of 10 min sonication in a water bath. The sample was then incubated at 80°C for 10 min, reduced (addition of 100 µl of 60 mM DTT and incubation at 65°C for 10 min) and alkylated (addition of 100 µl of 180 mM iodoacetamide and incubation at room temperature for 30 min in the dark). Trypsin (Sigma-Aldrich) was reconstituted in 50 mM acetic acid to a concentration of 0.2 µg/µl. Digestion was performed with 100 µl of trypsin at 37°C overnight. The RapiGest™ was removed from the sample by acidification (1 µl of trifluoroacetic acid and incubation at 37°C for 45 min) and centrifugation (15,000×g for 15 min). Peptide samples were divided into two tubes, one for global protein quantitation (equivalent of 100 µg protein) and one for phosphoproteome analysis (equivalent of 900 µg protein). Peptide samples for phosphoproteome analysis were enriched using titanium dioxide (TiO_2_) phosphopeptide enrichment and Clean-up Kit (Proteabio) following the manufacturers protocol.

Peptide mixtures from either whole lysates or phosphopeptide enriched samples were analysed by on-line nanoflow liquid chromatography using the nanoACQUITY-nLC system (Waters MS technologies, Manchester, UK) coupled to an LTQ-Orbitrap Velos (ThermoFisher Scientific, Bremen, Germany) mass spectrometer equipped with the manufacturer’s nanospray ion source. The analytical column (nanoACQUITY UPLC™ BEH130 C18 15 cm×75 µm, 1.7 µm capillary column) was maintained at 35°C and a flow-rate of 300 nl/min. The gradient consisted of 3–40% acetonitrile in 0.1% formic acid for 90 min then a ramp of 40–85% acetonitrile in 0.1% formic acid for 3 min. Full scan MS spectra (m/z range 300–2000) were acquired by the Orbitrap at a resolution of 30,000. Analysis was performed in data dependent mode. The top 20 most intense ions from MS1 scan (full MS) were selected for tandem MS by collision induced dissociation (CID) and all product spectra were acquired in the LTQ ion trap. Ion trap and orbitrap maximal injection times were set to 50 ms and 500 ms, respectively.

### Data analysis

Protein quantitation was achieved using intensity based label free protein quantitation [33], [34]. Thermo RAW files were imported into Progenesis LC–MS (version 4.1, Nonlinear Dynamics). Replicate runs were time-aligned using default settings and an auto-selected run as a reference. Peaks were picked by the software using default settings and filtered to include only peaks with a charge state of between +2 and +6. Peptide intensities of replicates were normalised against the reference run by Progenesis LC-MS. Spectral data were transformed to.mgf files with Progenesis LC–MS and exported for peptide identification using the PEAKS Studio 7 (Bioinformatics Solutions Inc.) search engine. Multiple search engine platform provided by PEAKS Studio named inChorus was used, which combines searching results from PEAKS DB (Bioinformatics Solutions Inc.), Mascot (Matrix Science), OMSSA (National Center for Biotechnology Information) and X!Tandem (Global Proteome Machine Organization). Tandem MS data were searched against a custom database that contained the common contamination and internal standards, PiroplasmaDB-3.0_TannulataAnkara_AnnotatedProteins and UniProt_Bos_taurus (Bovine) reviewed proteins. The search parameters were as follows; precursor mass tolerance was set to 10 ppm and fragment mass tolerance was set to 0.5 Da. One missed tryptic cleavage was permitted. Carbamidomethylation was set as a fixed modification and oxidation (M), phosphorylation at S, T, and Y set as variable modifications. The false discovery rates were set at 1% and at least two unique peptides were required for reporting protein identifications. The mass spectrometry proteomics data have been deposited to the ProteomeXchange Consortium (http://www.proteomexchange.org) via the PRIDE partner repository with the dataset identifier PXD000899 and DOI 10.6019/PXD000899 [35]. Phospho-epitope prediction was performed using Phosida online phosphorylation site database (PHOSIDA Posttranslational Modification Database, used on 30. March 2014: http://www.phosida.com) and NetPhosK [36] http://www.cbs.dtu.dk/services/NetPhosK/. The TMpred tool on the “SIB ExPASy Bioformatics Resources Portal” was used to predict protein topology of TaSP [37]. The sequence of p104 (TA08425) from TaC12 cells is published with the accession number XM_948006 [25]. Other *T. annulata* protein information was found using http://www.eupathdb.org (version 3 Feb 2014; ApiDB: integrated resources for the apicomplexan bioinformatics resource center. 2007 Jan; 35(NAR Database issue): D427-30 [38].

## Results and Discussion

### Immunofluorescence analysis (IFA) of *Theileria* infected cells reveals cell cycle-dependent phosphorylation of the schizont

**Figure 1:**
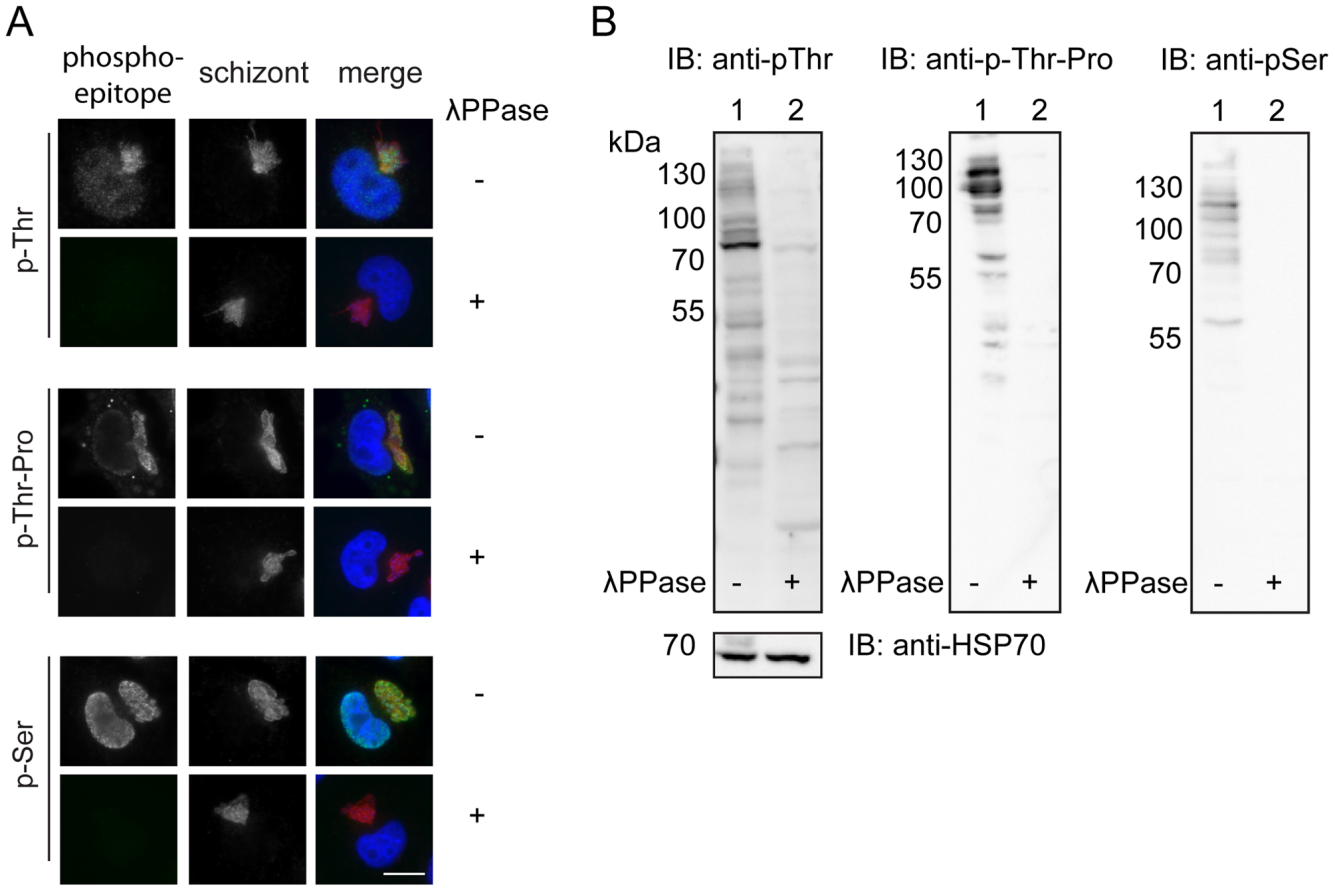
Validation of specificity of anti-p-Ser, p-Thr and p-Thr-Pro antibodies. A: TaC12 cells were fixed (with 4% PFA) and incubated overnight with or without λPPase at 30°C before labelling with anti-p-Ser, p-Thr and p-Thr-Pro antibodies. DNA is visualised with DAPI (blue). Merge: phospho-epitopes (green), anti-schizont (red), DAPI (blue). Scale bar represents 10 µm. B: Lysates of TaC12 cells were incubated overnight with (2) or without (1) λPPase at 30°C prior to Western blot analysis with anti p-Ser, pThr and p-Thr-Pro antibodies. As a control for equal loading the membranes were probed with mouse anti-*Theileria*-HSP70 antibody.

To investigate whether general phosphorylation of the *Theileria* schizont surface changes as the infected cell progresses through the cell cycle, we decided to analyse *T. annulata* infected macrophages (TaC12) by IFA using three different phospho-site specific antibodies: p-Thr, p-Thr-Pro and p-Ser. To verify the specificity of these phospho-antibodies, fixed cells were incubated with lambda protein phosphatase (λPPase) prior to IFA analysis (Figure 1A). λPPase treatment completely abolished the detection of p-Ser, p-Thr and p-Thr-Pro epitopes, while the signal obtained with a polyclonal anti-schizont antibody used to visualise the parasite was not affected by the treatment. Treatment of TaC12 cell lysates with λPPase resulted in a marked reduction in signal intensity with all phospho-specific antibodies by western blotting (Figure 1B). Thus we were satisfied that these antibodies specifically recognise phospho-epitopes in *Theileria*-infected cells.

**Figure 2:**
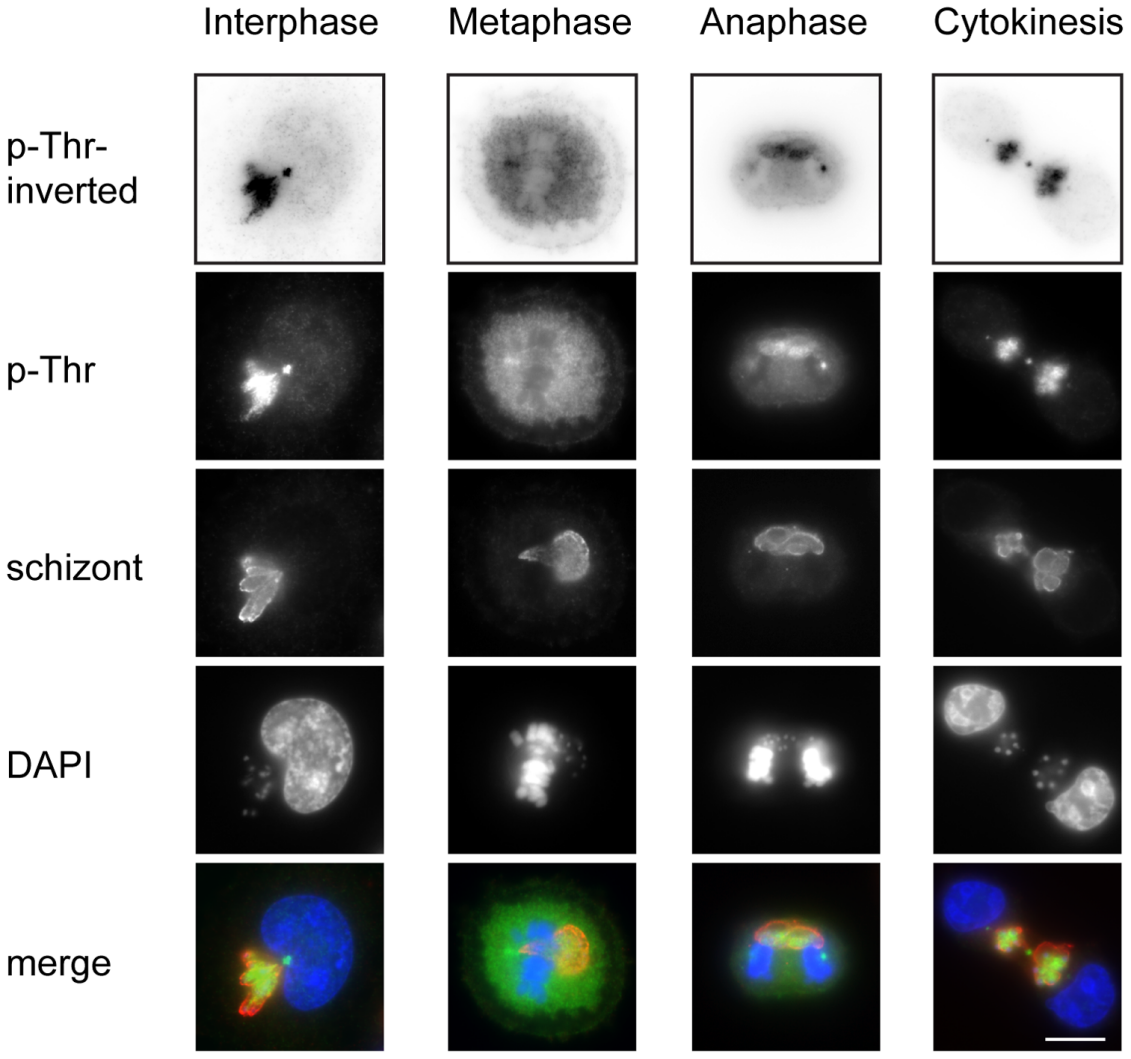
p-Thr epitopes are detected on the schizont during host cell interphase and cytokinesis. Unsynchronised TaC12 cells were fixed with methanol and representative cells from different cell cycle stages are shown. A p-Thr specific antibody was used to detect phosphorylation at threonine residues and the anti-schizont polyclonal antibody is used to label the parasite. DNA is labelled with DAPI. Merge: anti-pThr (green), anti-schizont (red), DAPI (blue). Scale bar represents 10 µm.

We detected p-Thr, p-Thr-Pro and p-Ser epitopes within the schizont and at the parasite surface during interphase (Figure 2 and S1A–C), while no distinct labelling of the parasite was observed with anti-p-Tyr antibodies (Figure S1D). p-Ser epitopes were additionally found to accumulate strongly at the “tips” of the schizont in many interphase cells (Figure S1C). It is well established that progression of cells into mitosis is accompanied by a massive increase in phosphorylation [39]–[41]. This was reflected in our own results, as the overall intensity of host cell phosphorylation increased during mitosis (figure S2). In prometaphase cells phosphorylation of the schizont was almost undetectable compared to the highly phosphorylated host cell cytoplasm (Figure S1 and S2). As the host cell progressed through metaphase and into anaphase phosphorylation of the schizont was observed once again, with phosphorylation on the schizont and within the host cell cytoplasm detected with p-Thr antibodies (Figure 2). As a control, unsynchronised uninfected bovine macrophages (BoMAC) were analysed with phoshpo-specific antibodies (Figure S3). As in TaC12 cells, pSer and pThr epitopes were detected in the nucleus in interphase, and dispersed within the cytoplasm during mitosis. The schizont was strongly phosphorylated during host cell telophase and cytokinesis, with some labelling of the surface obtained with anti-p-Thr-Pro antibodies (Figure S1 B). The p-Thr-Pro antibody used in this study is reported to recognise threonines and some serines that are specifically targeted by proline-directed kinases, namely cyclin-dependent kinases (CDKs) with a consensus motif of S/T-P-X-K/R, and mitogen-activated protein kinases (MAPKs) that recognise P-X-S/T-P motifs [42]. Our data therefore indicate the presence of CDK and/or MAPK substrates on the parasite surface during host cell interphase, telophase and cytokinesis.

**Figure 3:**
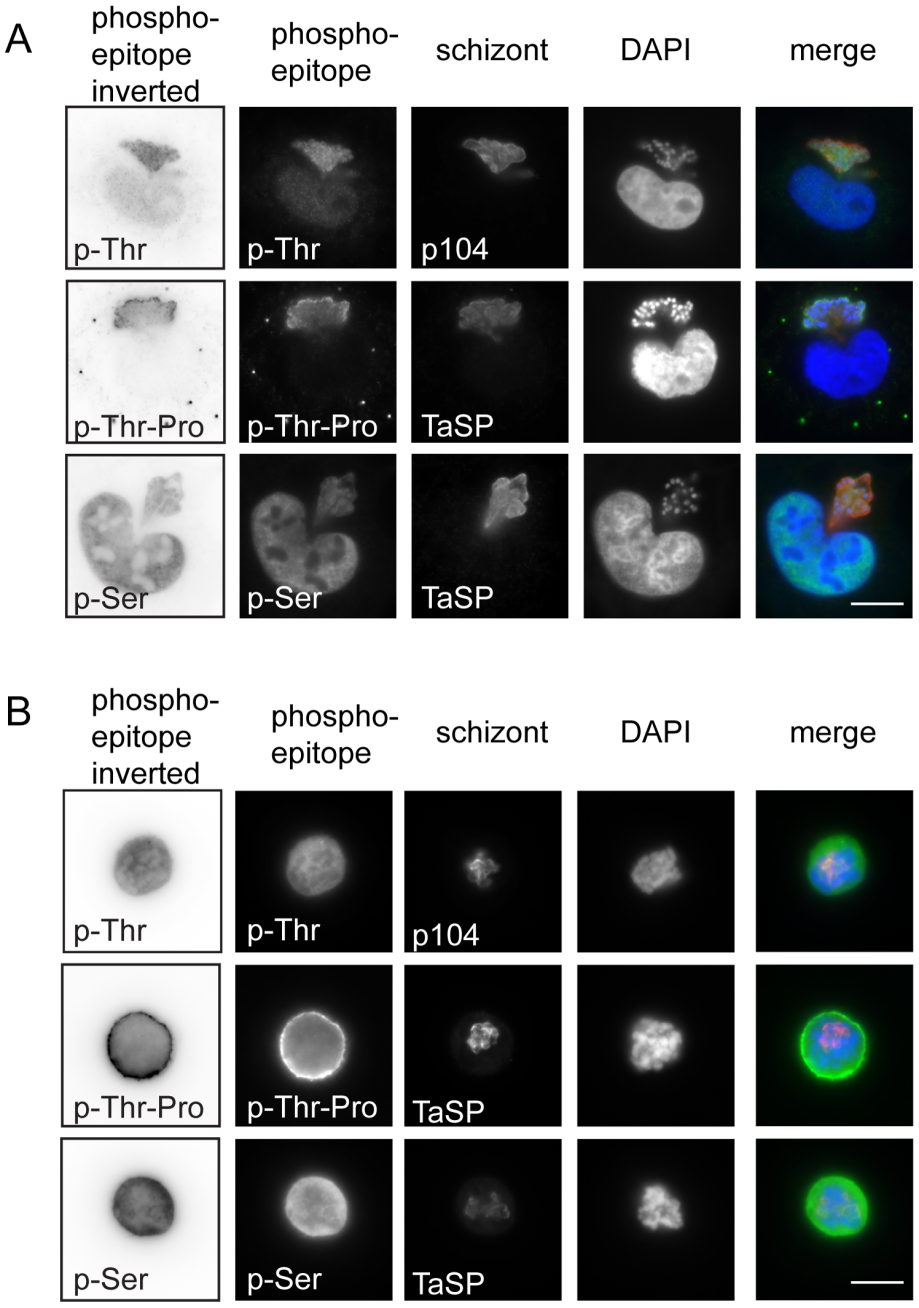
Synchronisation of TaC12 cells in S- and M-phase. TaC12 were treated with thymidine for 24 hours or nocodazole for 16 hours to synchronise cells in S-phase or mitosis. Synchronised cells were fixed with 4% PFA and analysed with anti-p-Thr, anti-p-Thr-Pro and anti-p-Ser antibodies. The parasite was detected with anti-p104 or TaSP antibodies and DNA is visualised with DAPI. Merge: phospho-epitopes (green), schizont (red), DAPI (blue). A: Thymidine synchronised TaC12 cells in S-phase. B: Nocodazole synchronised TaC12 cells in mitosis. Scale bar represents 10 µm.

To investigate the cell cycle-dependent differences in schizont phosphorylation in more detail, we treated TaC12 cells with thymidine to synchronise them in S-phase, and with nocodazole to synchronise them in mitosis as described [24]. Phosphorylated histone H3 was strongly detected by both Western blotting and IFA during mitosis and was absent during S-phase, confirming successful cell synchronisation (Figure S4). Consistent with our results in unsynchronised cells (Figures 2 and S1), the schizont was distinctly labelled with p-Ser, p-Thr and p-Thr-Pro antibodies during S-phase, while less clear phosphorylation of the parasite could be detected in cells blocked in mitosis (Figure 3). When TaC12 cells were released for 6 hours from a thymidine block and allowed to accumulate in G2 phase [24], p-Thr epitopes were mainly detected within the parasite cytoplasm and in parasite nuclei (data not shown). These initial analyses indicate that phosphorylation of the schizont varies as the host cell progresses through the cell cycle, and is compatible with the hypothesis that differential phosphorylation of substrates at the parasite surface might contribute to cell cycle-dependent host-pathogen interactions. Considering the phosphorylation of the schizont surface during interphase, we next wanted to identify parasite phosphoproteins by mass spectrometry.

### Enrichment of schizonts from cells synchronised in S-phase and mitosis

**Figure 4:**
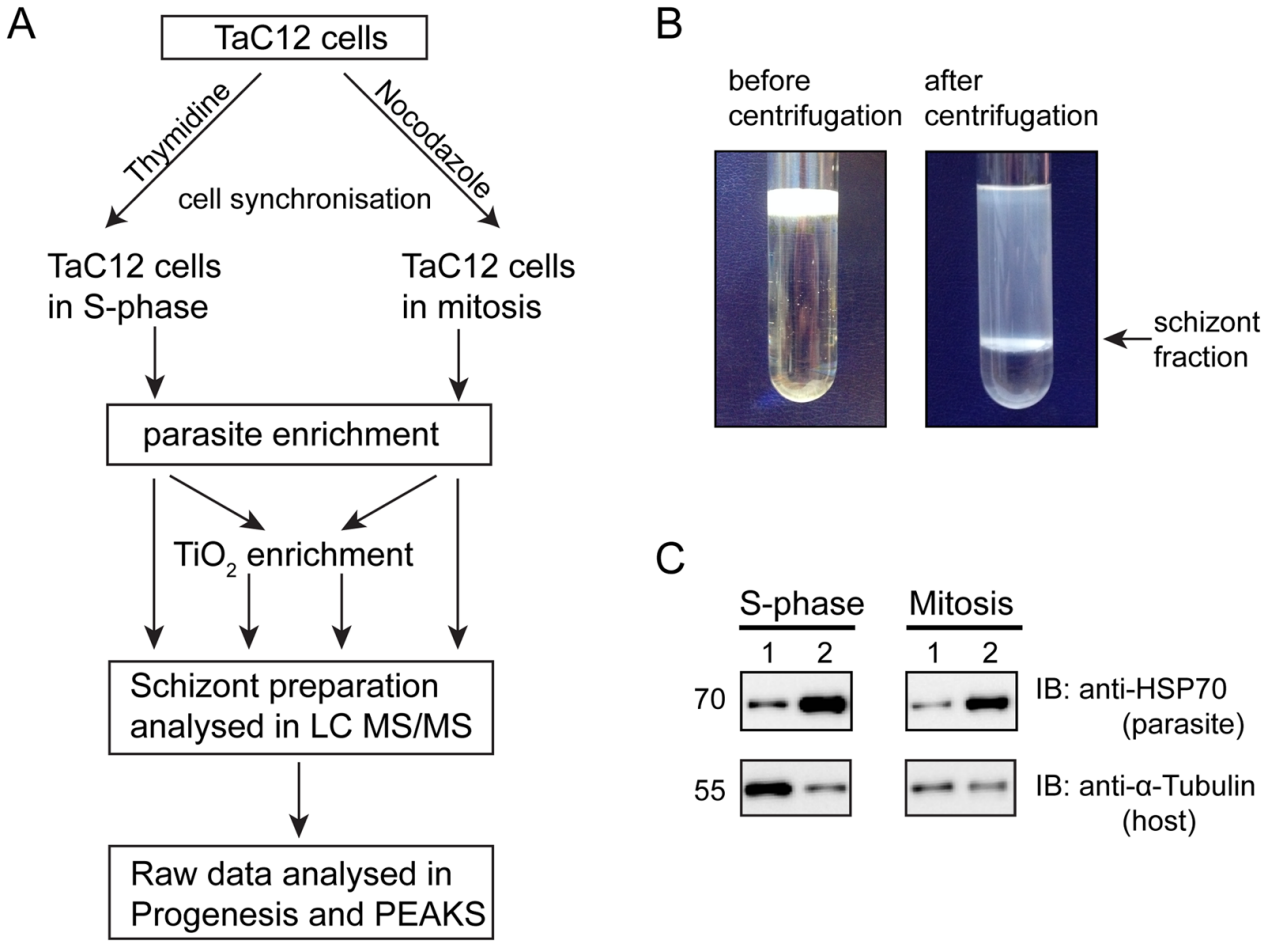
Enrichment of schizonts from cells synchronised in S-phase or M-phase. A: Overview of the workflow: TaC12 cells were synchronised in S-phase (thymidine block) or mitosis (nocodazole block). Schizonts were enriched, lysed, and analysed directly by LC MS/MS or after TiO_2_ enrichment of phosphopeptides. Raw data were analysed using Progenesis and PEAKS. B: Schizonts were enriched using a Nycodenz step gradient. The sample is shown before (left) and after (right) centrifugation. The fraction containing enriched schizonts is indicated with an arrow. C: Western blot analysis of whole cell lysates (1) compared to purified schizonts (2). Equal amounts of protein from whole TaC12 lysates and enriched schizonts purified from S-phase and mitosis synchronised cells were subjected to Western blotting using an anti-*Theileria*-HSP70 antiserum to detect the parasite and anti-tubulin to detect host cell tubulin.

To facilitate a comparative phosphoproteome analysis we decided to purify schizonts from both S-phase and M-phase synchronised cells. Since a published protocol [27] was very inefficient for the isolation of schizonts from synchronised mitotic cells (data not shown), we tested several modifications to the protocol, and found that a low-speed Nycodenz step gradient could be used to separate schizonts from mitotic host cell debris (Figure 4). We found that these modifications enabled reproducible enrichment of parasites from M- and S-phase cells with a reduced purification time. The successful enrichment of parasites was verified by Western blotting with anti-*Theileria* Hsp70 and anti-(host cell) tubulin antibodies (Figure 4C) and IFA (data not shown).

**Figure 5:**
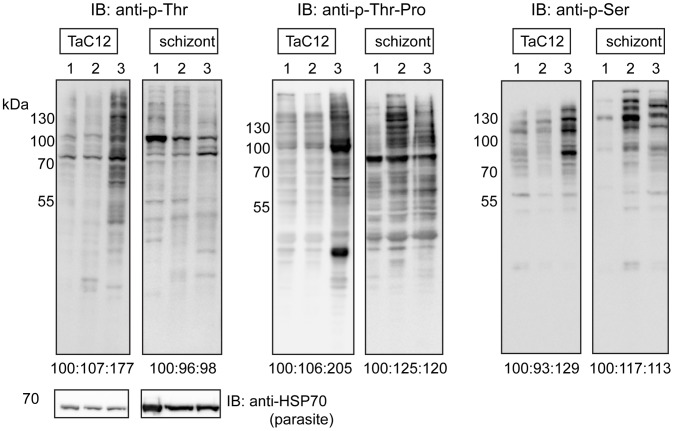
Western blot analysis of whole cell and purified schizont lysates with anti-phospho-antibodies. The phosphorylation pattern of asynchronous (lane 1) and synchronised TaC12 cells in S-phase (2) and mitosis (3) and corresponding samples with enriched schizonts were analysed with anti-p-Thr, anti-p-Thr-Pro and anti-p-Ser antibodies by Western blot. An equal amount of protein was loaded in each lane. Anti-*Theileria* HSP70 was used as a loading control. Signal intensity for each lane was quantified and the ratios are indicated underneath (also depicted in figure S5).

Whole TaC12 cells and enriched parasite samples were lysed and an equal amount of protein was subjected to Western blot analysis. This confirmed that phoshpo-epitopes were preserved following schizont enrichment, and allowed us to analyse changes in phosphorylation patterns between S-phase or M-phase synchronised whole cells (TaC12) and enriched parasites (schizonts) (figure 5). In whole cell lysates of TaC12 cells the signal detected with all three phospho-antibodies increased in mitotic cells (Figure 5 lane 3 in each case and figure S5) compared to unsynchronised cells or those blocked in S-phase (lanes 1 and 2). This was as expected [39]–[41], and confirmed our IFA data that showed that overall phosphorylation of the host cell increased during mitosis (Figure S2). Conversely, phospho-epitopes were readily detected in schizont lysates enriched from both S-phase and mitotic cells. This indicates that the schizont is phosphorylated in both S-phase and in mitosis, although we cannot exclude the possibility that some bovine phospho-peptides were also present following parasite enrichment. These data support our observations made with IFA that in S-phase cells, the level of schizont phosphorylation is high in comparison to host cell phosphorylation (Figure S2).

The ability to isolate *Theileria* schizonts from its host cell has provided an invaluable tool in the field of *Theileria* research, and has facilitated high resolution imaging of the parasite surface [43] as well as a recent proteome analysis of the schizont [21]. While ultracentrifugation with a percoll gradient can be used to produce highly pure preparations of schizonts [27], we recommend the use of the rapid method presented here, which requires minimal handling, for enrichment of schizont proteins for subsequent biochemical analysis. This method is particularly useful where prior synchronisation of the host cell is desired.

### Label-free mass spectrometry analysis of *Theileria* schizonts from synchronised cells

**Figure 6:**
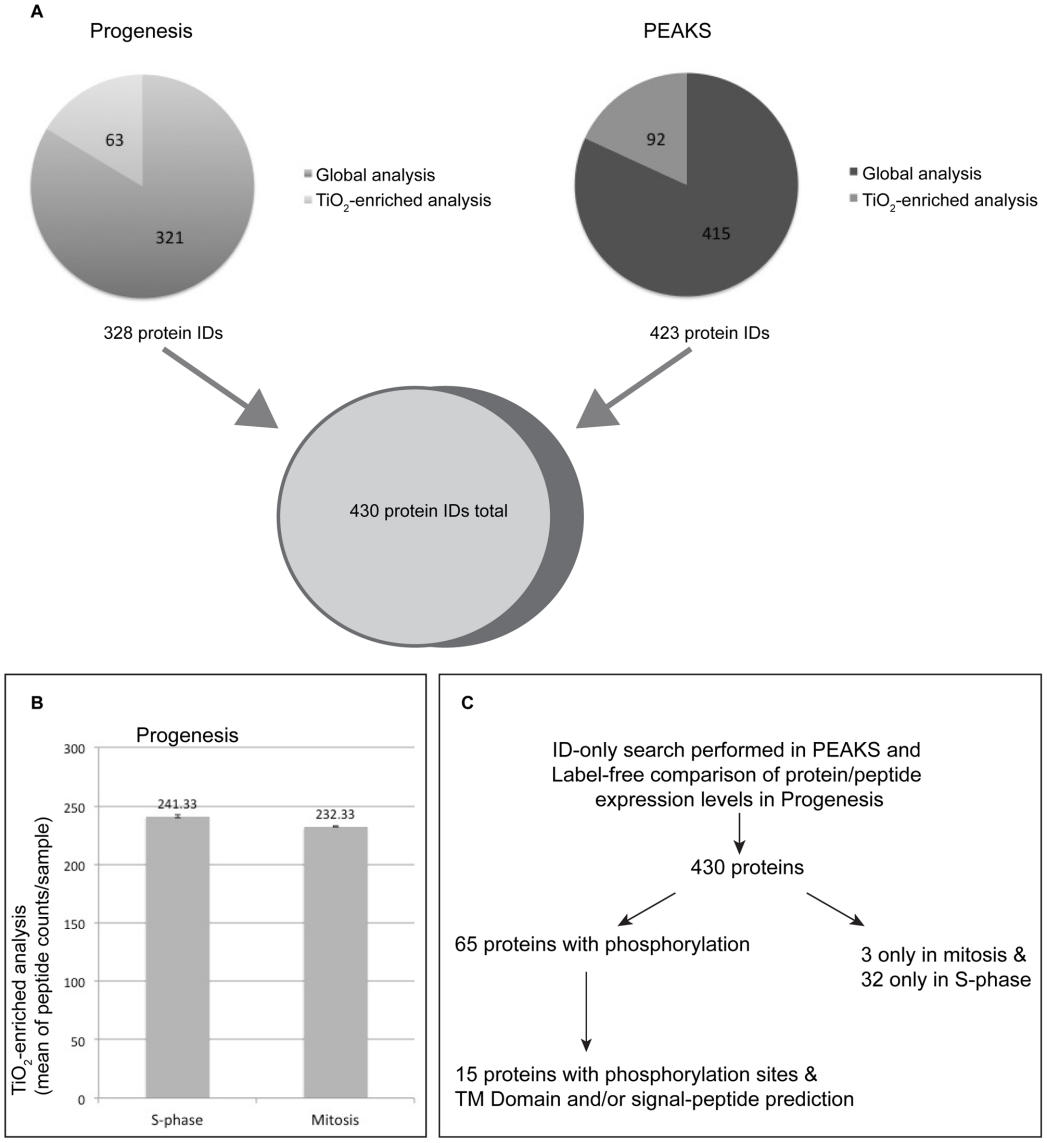
Overview of the mass spectrometry results. A: Identified proteins using Progenesis and PEAKS. B: Mean peptide counts per sample corresponding to *T. annulata* proteins identified using Progenesis following TiO_2_ enrichment of S-phase (n = 3) or mitotic (n = 3) samples. C. Overview of identified *T. annulata* proteins.

For the mass spectrometry analysis *T. annulata* parasites were enriched from host cells synchronised in S-phase or M-phase as described (work flow summarised in Figure 4A). For each condition, three replicates were prepared. Each sample was split into two; one for direct analysis by LC MS/MS (Global) while the other was subjected to phospho-peptide enrichment (TiO_2_ enrichment). Three replicates of both M-phase and S-phase were run simultaneously and the raw data were analysed with Progenesis LC-MS (Nonlinear Dynamics) and PEAKS Studio 7 (Bioinformatics Solutions Inc.). In total we detected 1317 proteins, of which 430 are of *T. annulata* origin, and 887 are bovine (Figure 6, Tables S1 and S7). 31 *Theileria* proteins were detected in this study that were absent from a previous *Theileria* proteomic analysis [21]. While most of the *Theileria* proteins were detected in all replicate samples, three proteins were detected only in M-phase, and 32 were found only in S-phase samples (Table S2).

In the comparative search performed with Progenesis, the ion intensities recorded for all samples (six “Global” samples or six TiO_2_-enriched samples) were compared. With this search the abundance of 328 *Theileria* proteins was calculated and compared between S-phase and mitotic samples. Of these, the relative abundance of 32 proteins could be compared in a statistically significant manner (p-value <0.05) (Table S3). All of the schizont proteins that were detected with higher abundance from S-phase samples (max fold change >1.5) are involved in protein transcription and translation. For example, the hypothetical protein TA05730, detected with a 41-fold higher abundance in samples from S-phase cells, belongs to the ribosomal protein L1 superfamily (Superfamily SSF56808). On the other hand, expression of parasite histones was found to be up to 7 fold higher in schizonts enriched from M-phase blocked cells when compared to those from S-phase cells (Table S3). In general histone synthesis coincides with DNA synthesis, and an increase in histone expression has been described during S phase in mammalian cells [44]. It was previously shown that *T. parva* initiates DNA synthesis when the host cell is in mitosis [45], although this has never been tested for *T. annulata*. We therefore analysed the incorporation of the thymidine analogue 5-bromo-2-deoxyuridine (BrdU) into host and parasite nuclei while cells were released from S-phase-block. We found that the incorporation of BrdU into parasite nuclei correlated inversely with host cell DNA synthesis and increased as the host cells progressed through mitosis (Figure S6), consistent with the observations made for *T. parva* [45], and providing an explanation for the increase in parasite histone expression observed during host cell mitosis.

We identified 124 bovine and 65 schizont proteins with at least one phosphorylation site (Table S4, S5, S7). Because our study was designed to enrich for schizont proteins and to reduce bovine proteins from our samples, we dare not speculate too much upon the bovine phospho-proteins identified in our study. Therefore we make no attempt to draw conclusions regarding the potential cell cycle dependent or *Theileria*-dependent phosphorylation of bovine proteins. However, because no phospho-proteome analysis of *Theileria*-infected cells has been published, we provide our data as a Table S7 for those interested. Of the 65 newly identified *Theileria* phospho-proteins, 27 are annotated in EupathDB as hypothetical proteins. A number of *Theileria* encoded enzymes were identified in our analysis, including a putative glycogen synthase kinase (TA02550), and a serine-threonine protein kinase (TA19110). We were particularly interested in phosphorylated schizont proteins that have the potential to interact with the host cell and for this reason we focused on proteins that are predicted to be expressed on the surface of the parasite or to be secreted into the host cell cytoplasm. Of the 65 phosphorylated schizont proteins identified, 15 possess a predicted signal peptide, transmembrane-domain(s) or a GPI anchor sequence (Table 1).

**Table 1:** List of phosphorylated proteins with a predicted transmembrane-domain and/or a signal peptide.

Accession	Description	Peptide counts	Sequence	Phospho-epitope	Kinase prediction	Highest detection in S or M phase	# TM Domains	SignalP Prediction
TA03495	Hypothetical	2	SGVLESNLSPKLTS	S741	PKC		2	
				S744	CK1, CDK1, MAPK			
				S1050				
			TTRLNSNISSPVNVP	S1054	CK1, GSK3, CAMK2, PKA, PKG, PKC, CDK5			
TA05145	Hypothetical	4	ELRIDSSKTLP	S249	CAMK2, PKA		10	
TA05215	Hypothetical	1	FRKSFSDVRLA	S1045			1	
TA05455	ABC transporter	2		S1352	PKA, PKD, Aurora, CHK1		9	
			INKRVSI**S**PECL**S**PSNQR	**S1354**		Mitosis		
				**S1359**	CDK5			
TA08425	P104	18	DELVMSPIPTT	S25	MAPK, NEK6, GSK3	S phase		
			PKRPV**S**PQRPV**S**PRRPE	**S601**	PKA, CDK2, CAMK2, MAPK, CDK1	S phase		
				**S607**	CDK2, CAMK2, MAPK, PKC, CDK1			
			PKSPKSPKRPE	S622	CK1, CDK2, MAPK, CDK1, PKC			
			PKSPKSPKVPF	S652	CK1, MAPK, CDK1, GSK3			
			KKRRRSDGLAL	S769	PKA, CAMK2, PKG			
			DGLALSTTDLESE	S775	NEK6			
				T776	CK2			
				T777	CK2, NEK6, CDK1	S phase		
			IVTMKR**S**K**S**FDDLTTVREK	**S800**	CK1, NIMA, PKA, PKG	S phase		
				**S802**	PKA, CAMK2, CHK1			
				T807	CK2, PKC			
			VDDDGTEADDE	T829	CK2			
			EDTHPSKEKHL	T839				
TA17315	TaSP	6	SHPAR**S** S**S**FSRIN	**S303**	GSK3	S phase	3	Yes
				S304	PKA, PKC, CDK1			
				**S305**	CAMK2, AKT, CDK1	S phase		
TA14990	Cation transporting ATPase	1	VPGNI**S**GDNIF	**S311**	CK2	S phase	9	
TA17300	cdp-diacylglycerol synthase	1	INRASSSQNSL	S101	GSK3, CAMK2, PKD, CHK1		7	
TA05190	Hypothetical	1	CDIILSIDDKN	S802	CK2		2	
TA14370	Hypothetical	9	KSSTG**S**PRSKM	**S21**	CK1, CDK1, MAPK, PKC, GSK3	Mitosis	1	
			QNAVSSGDESDIST	S253	CK2, GSK3,			
				S247	CK1, DNAPK, ATM, PKC			
TA19720	Hypothetical	2	GKALD**S**DDEDF	**S423**	CK2	S phase	1	
TA14665	Hypothetical	1	LARR**S**SSQTGFV	**S247**	PKA, CAMK2, PKD, CHK1	Mitosis	1	Yes
TA19115	SfiI-subtelomeric fragment related protein	1	SAKPGYSIIKV	Y1891	ALK		0	Yes/signal anchor
TA11580	dynamin-like protein	5	PKFEASPKLLI	S543	CDK1, MAPK		0	Yes
			KNTEASPKTIM	S567	CDK1			
TA06395	Hypothetical	7	SGLHESSCNSTPREG	S27			0	Yes
				S30	CK1, PKC, CDK1			

Phospho-epitopes indicated in bold were those found more abundantly in samples from M- or S-phase (p<0.05).

Our analysis revealed phosphorylation on the hypothetical protein TA14665 (Table 1) that possesses a predicted signal peptide, nuclear localisation signal and a PEST motif, and has as such been highlighted as a candidate manipulator of host cell phenotype [23]. Another potentially interesting phosphorylated protein found in our analysis is TA19115 (*Sfi*l-subtelomeric fragment related protein family member); a 272 kDa protein that belongs to a *Theileria*-specific, hypervariable sub-telomeric repeat family and possesses 21 FAINT (Frequently Associated in *Theileria*) domains [22]. While the function of FAINT domains remains unknown, they are particularly abundant in secreted *Theileria* proteins and, as such, have been predicted to play a role in host cell modification [22], [46]. Based on the presence of a predicted signal peptide and the multiple FAINT domains, this protein can be considered a candidate for host cell modification. Analysis with kinase prediction software (Phosida) indicates that the receptor tyrosine kinase ALK (Anaplastic lymphoma kinase) has the potential to phosphorylate TA19115 at Y1891.

### Cell cycle-dependent phosphorylation of schizont surface antigens, TaSP and p104

**Figure 7:**
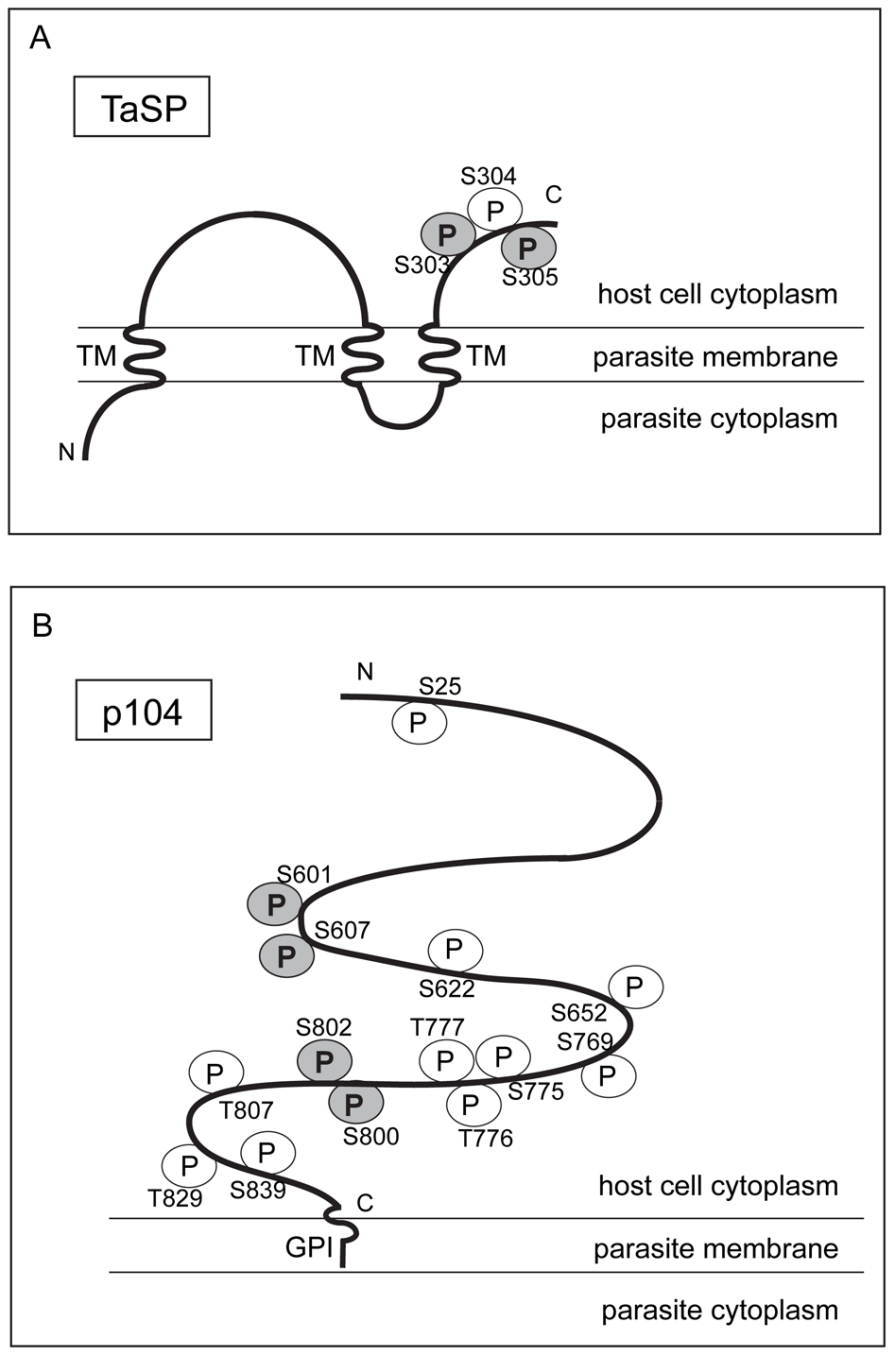
Schematic overview showing all phosphorylated sites detected on A) TaSP (TA17315) and B) p104 (TA08425). Detected phosphorylation sites are indicated with the amino acid number. White circles (P) represent phosphorylation sites detected in both mitotic and S-phase samples. Grey circles (P) represent phosphorylation sites with a significant difference in abundance between S-phase and mitotic samples. TaSP: schematic topology as predicted by TMpred: 1^st^ TM: 3-21 aa; 2^nd^ TM: 205-223 aa and 3^rd^ TM 262-288 aa. Two phosphorylated serines (S303 and S305) in the C-terminal domain were more highly phosphorylated in S-phase schizont-samples (p<0.01). p104 is predicted to have a GPI-anchor (GPI). Phosphorylation of four serines (S601, S607, S800 and S802) was significantly increased in S-phase when compared to mitosis.

We found that most of the phosphorylated peptides that we identified following TiO_2_ enrichment were found in all samples (Figure 6B). However, in several cases, the peptide abundances were significantly different between samples. Two well-characterised schizont surface proteins, TaSP (TA17315) and p104 (TA08425) were found to be differentially phosphorylated in host cell S- and M-phase. TaSP is an abundant surface protein that was reported to interact with host cell alpha tubulin, and thus contributes to the association of microtubules with the schizont surface [28]. Three phosphorylated serines (S303, S304 and S305) were identified in the C-terminal domain that is exposed to the host cell cytoplasm (Figure 7, Tables 1, S5 and S6,) [47]. Two of these sites (S303 and S305) were phosphorylated with up to a 350-fold increase in schizonts enriched from S-phase cells compared to M-phase cells (Figure S7A). Prediction tools suggest that these sites could be targets of Akt, PKA or glycogen synthase kinase 3 (GSK3) (Table 1). The activity of both Akt and PKA in *Theileria*-infected cells has been reported and linked to the proliferation or survival of infected cells respectively [16], [17].

Fourteen phosphorylation sites were detected on p104 (TA08425), a schizont surface protein that possesses four FAINT domains and a C-terminal domain that is highly disordered and rich in prolines, serines and basic residues. The C-terminal domain of p104 encompasses many “PxxP” Src homology 3 (SH3)-binding motifs that have the potential to interact with multiple binding partners, and a verified “SxIP” motif that mediates the cell cycle-dependent interaction of the parasite with the host cell microtubule plus end tracking protein (+TIP) EB1 [25]. Of the 14 phosphorylated residues identified on p104, several are potential targets of PKA (consensus motif R-R/K-X-S/T) or CK2 (S/T-D/E-X-E/D) (Table 1), both of which are constitutively activated in *Theileria* infected cells [16], [20]. Four of the 14 phosphorylated residues (S601, S607, S800 and S802) are more highly phosphorylated during S-phase (p<0.0013). On one peptide that spans S601 and S607, a 9000-fold increase in phosphorylation was detected in parasites enriched from S-phase cells compared to M-phase cells (Figures 7 and S7B, Table S6) (p = 0.0006). Importantly, phosphorylation sites that were strongly increased in S-phase samples have a significant p-value, while the total protein expression levels of p104 did not change between S-phase and mitosis (Figure S7C, Table S3).

S601 and S607 of p104 fulfil the consensus motif for both CDKs (S/T-P-X-K/R) and MAPKs (P-X-S/T-P) (Table 1). Specificity of MAPK and CDK activity is further regulated by the presence of docking motifs on target substrates, and in this context it is important to note that in addition to phosphorylated motifs, several MAPK docking motifs (R/K)_1–2_,-(X)_2–6_-Φ-X-Φ) (where Φ represents a hydrophobic residue) and cyclin-docking motifs (R/K-X-L) are present within p104 [42], [48]. In addition to being present within p104, several putative CDK or MAPK sites were phosphorylated within the 15 potentially surface expressed proteins described in Table 1. The identification of phosphorylated CDK/MAPK motifs on surface proteins by mass spectrometry is consistent with the detection of p-Thr-Pro epitopes by IFA on the parasite surface, and indicates that proline-directed kinases phosphorylate the schizont during host cell S-phase (Figure S1). It has been shown by several groups that of the MAPK family, JNK is constitutively activated in *Theileria*-transformed cells while extracellular signal-related kinase 2 (ERK-2) and p38 are not active [9]–[11], [15]. It could therefore be of interest to investigate whether *Theileria* surface expressed proteins are substrates of JNK, and if so, whether this might contribute to maintaining the transformed phenotype.

In our previous work we showed by gel shift assay that endogenous p104 is highly phosphorylated in unsynchronised cell cultures (which mainly consist of cells in G0-G1-phase, Figure S4A), with a slight increase in the overall phosphorylation of p104 detected in mitotic cells [25]. Because the interaction of many plus end tracking proteins with EB1 is regulated by phosphorylation in the vicinity of the SxIP motif, we focused in detail on a short fragment (p104_521–634_) that encompasses the EB1-binding domain and 21 potentially phosphorylated sites (dashed underlined in Figure S4). We showed that this short fragment, like the endogenous protein, is phosphorylated and that in this case the “up-shift” observed in mitotic cells compared to unsynchronised cells was incredibly striking. This indicated to us that this region is subjected to extensive cell cycle-dependent regulation of phosphorylation. We went on to show that CDK1 activity is partially, but not completely, responsible for phosphorylating p104 during mitosis. In our current work, we identified three phosphorylated serines within this fragment, and show that two of them (S601 and S607) are in fact highly phosphorylated during S-phase (Figure 7 and S7, Table S6). Together, the data from our present and previous work indicate that p104 is extensively phosphorylated, and that distinct residues are differentially phosphorylated in a cell cycle-dependent manner. Considering the multiple protein-binding domains in p104, it will be of interest to further investigate the putative interaction of p104 with other host cell proteins, and the cell cycle-dependent changes in phosphorylation described here should be taken into account.

### Concluding remarks

The work presented here represents the first, albeit partial, analysis of phosphorylation events on *T. annulata* schizont proteins. In particular, we identified cell cycle-dependent phosphorylation of the abundant surface proteins TaSP and p104 that have the potential to be involved in host-parasite interactions, or even signal-transduction pathways involved in the transformation process. These data certainly warrant further mass spectrometry based investigations of phosphorylation events in *Theileria* infected cells. In particular the application of multiple fractionation steps to increase sequence coverage is likely to be of value. A recent comparative microarray analysis between non-infected, *Theileria*-infected, and *Theileria*- cured bovine lymphosarcoma cells revealed over 3000 *Theileria*-dependent changes in host cell gene expression, in particular genes encoding transcription factors and modifiers of chromatin [49]. A comparative phospho-proteomic analysis of host cell proteins between *Theileria*-infected, non-infected and cured cell lines is likely to provide insights into this fascinating phenomena of reversible transformation that could be of high impact in the wider field of signal transduction.

## Supporting Information

Figure S1 p-Thr, p-Ser and p-Thr-Pro epitopes are detected on the schizont during host cell interphase, mitosis and cytokinesis. A-D: Unsynchronised TaC12 cells were fixed with 4% PFA and labelled with specific antibodies detecting A: p-Thr, B: p-Thr-Pro, C: p-Ser and D: p-Tyr epitopes. An anti-schizont polyclonal antibody is used to label the schizont and DNA is labelled with DAPI. Merge: anti-phospho-epitope (green), anti-schizont (red) and DAPI (blue). Scale bar represents 10 µm.
(TIF)

Figure S2 Quantified fluorescence intensity on parasite or in host cell cytoplasm. A: Immunofluorescence signal of unsynchronised TaC12 cells in S-phase or mitosis were analysed using ImageJ. Images were captured using the same exposure time for each cell. Mean fluorescence intensity was calculated in an area at the parasite or in the host cell cytoplasm (Yellow circles). A representative image following p-Thr labelling is show. B: Comparison of the mean fluorescence intensity of the phospho-epitope specific antibodies p-Thr, pThr-Pro and p-Ser at the parasite and in the host cell cytoplasm in mitosis and in S-phase. Statistically significant differences were observed between S-phase and mitosis samples for the host cell cytoplasm intensity for each antibody used (pThr p = 7×10^−8^, pThr-Pro p = 0.0014, Ser p = 0.0004), and between parasite and host cell in S-phase samples (pThr p = 1.7×10^−9^, pThr-Pro p = 2.7×10^−5^, pSer p = 3×10^−12^). **** denotes a p value <0.0001, while *** denotes a p value between 0.001 and 0.0001 (unpaired t-test, two-tailed).
(TIF)

Figure S3 Detection of p-Thr, p-Ser and p-Thr-Pro epitopes in uninfected bovine macrophages (BoMAC) during host cell interphase and mitosis
(TIF)

Figure S4 Synchronisation of TaC12 cells in S- or M-phase. A: Asynchronous TaC12 cells and cells incubated for 24 h in thymidine (S-phase) or 16 h in nocodazole (M-phase) were fixed in 80% ethanol and the DNA content was labelled with propidium iodide prior to FACS analysis. B: Lysates from TaC12 cells (unsynchronised, S-phase or M-phase) were analysed by Western blot using anti-cyclin-A and anti-p-Histone H3 antibodies. As a loading control anti-*Theileria*-HSP70 was used. C: Following synchronisation TaC12 cells were fixed with 4% PFA and labelled with a polyclonal anti-schizont antibody and anti-p-Histone H3. DNA was visualised with DAPI. Merge: anti-p-Histone3 (green), anti-schizont (red) and DAPI (blue). Scale bar represents 10 µm.
(TIF)

Figure S5 Relative signal intensity following western blotting of TaC12 and schizont lysates with anti-p-Thr, p-Thr-Pro and p-Ser antibodies. Relative intensities were measured using ImageJ, and correspond to the western blots shown in figure 5.
(TIF)

Figure S6 Parasite DNA replication occurs as the host cell progresses through mitosis. A: TaC12 cells were synchronised in S-phase with thymidine treatment, and released into fresh medium. BrdU (10 µM) was added to the culture 2 hours prior to analysis at 6, 8, 10 and 12 hours after thymidine release. Cells were fixed with 4% PFA and BrdU incorporation into host (H) and parasite (P) nuclei was analysed by IFA. Quantification of cells that had incorporated no BrdU (H−/P−) are excluded from the graph for clarity. n = 70−120 cells per time point. B: Representative images of cells at 6 hours (left) and 12 hours (right) post thymidine release are shown. The schizont is labelled green and BrdU is red. Scale bar represents 10 µm.
(TIF)

Figure S7 TaSP (TA17315) and p104 (TA08425) protein and phosphopeptide abundances. A: Three phosphorylation-sites were detected in TaSP. Two phosphorylated residues were found with a higher abundance in S-phase (p<0.01). Data were analysed with Progenesis. The max fold change for each significant (p<005) paired scanning event for peptides that were differentially detected between S- and M-phase are shown in a table (data extracted from table S6). The normalised peptide abundance for peptide SSSFSRINEDCC in S-phase and mitosis samples is presented as a histogram (consensus of all paired scanning events). B: 14 phospho-sites in p104 were detected (bold in the protein-sequence). Two detected phosphorylated peptides (corresponding to four phospho-sites) were more abundantly detected in S-phase samples (p<0.002). The max fold change for each significant (p<005) paired scanning event for peptides that were differentially detected between S- and M-phase are shown (data extracted from table S6). The normalised peptide abundance for peptides RPVSPQRPVSPR and SKSFDDLTTVR in S-phase and mitosis samples (consensus of all paired scanning events) is shown. The sequence corresponding to p104_521−634_ is underlined (dashed). C: Protein abundance of the *Theileria* surface proteins p104 and TaSP in the “Global”-analysis using Progenesis.
(DOCX)

Table S1 All *T. annulata* proteins detected by LC MS/MS are listed with the corresponding protein information.
(XLSX)

Table S2 List of all *T. annulata* proteins detected only from mitotic or S-phase synchronized samples.
(XLSX)

Table S3 List of all *T. annulata* proteins for which the relative abundance could be compared between all six samples (p<0.05; Progenesis).
(XLSX)

Table S4 List of all phosphoepitopes detected, with the corresponding protein ID and description (“Global” analysis and TiO2 enriched samples). One peptide hit proteins were also included and are indicated in the list.
(XLSX)

Table S5 List of all phosphopeptides detected using PEAKS and Progenesis, with the corresponding protein ID and description (“Global” analysis and TiO_2_ enriched samples).
(XLSX)

Table S6 List of all detected *T. annulata* phosphorylated peptides for which the relative abundance could be compared (p<0.05) between all the 6 samples (“Global” analysis and TiO_2_ enriched samples; analysed using Progenesis).
(XLSX)

Table S7 List of all detected bovine proteins and phosphorylated peptides. Proteins containing at least one phosphorylated residue are listed.
(XLSX)
