# Supplementary figures and images for: Cell Cycle-Dependent Phosphorylation of Theileria annulata Schizont Surface Proteins
Source: PLoS One. 2014 Jul 31;9(7):e103821. doi: 10.1371/journal.pone.0103821 (PMC4117643; doi:10.1371/journal.pone.0103821)

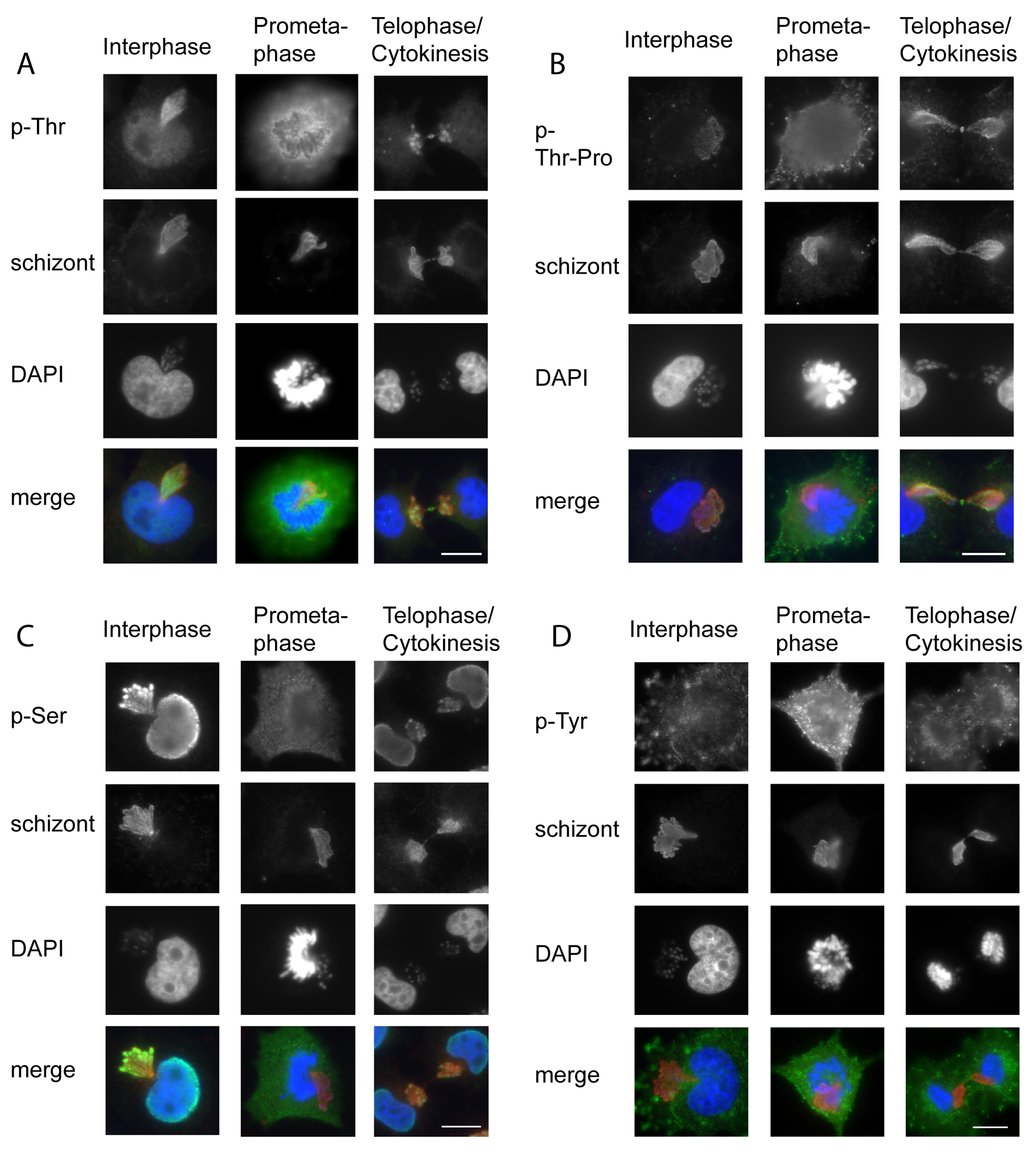

Supplement: Figure S1 — p-Thr, p-Ser and p-Thr-Pro epitopes are detected on the schizont during host cell interphase, mitosis and cytokinesis. A-D: Unsynchronised TaC12 cells were fixed with 4% PFA and labelled with specific antibodies detecting A: p-Thr, B: p-Thr-Pro, C: p-Ser and D: p-Tyr epitopes. An anti-schizont polyclonal antibody is used to label the schizont and DNA is labelled with DAPI. Merge: anti-phospho-epitope (green), anti-schizont (red) and DAPI (blue). Scale bar represents 10 µm. (TIF) [file pone.0103821.s001.tif]

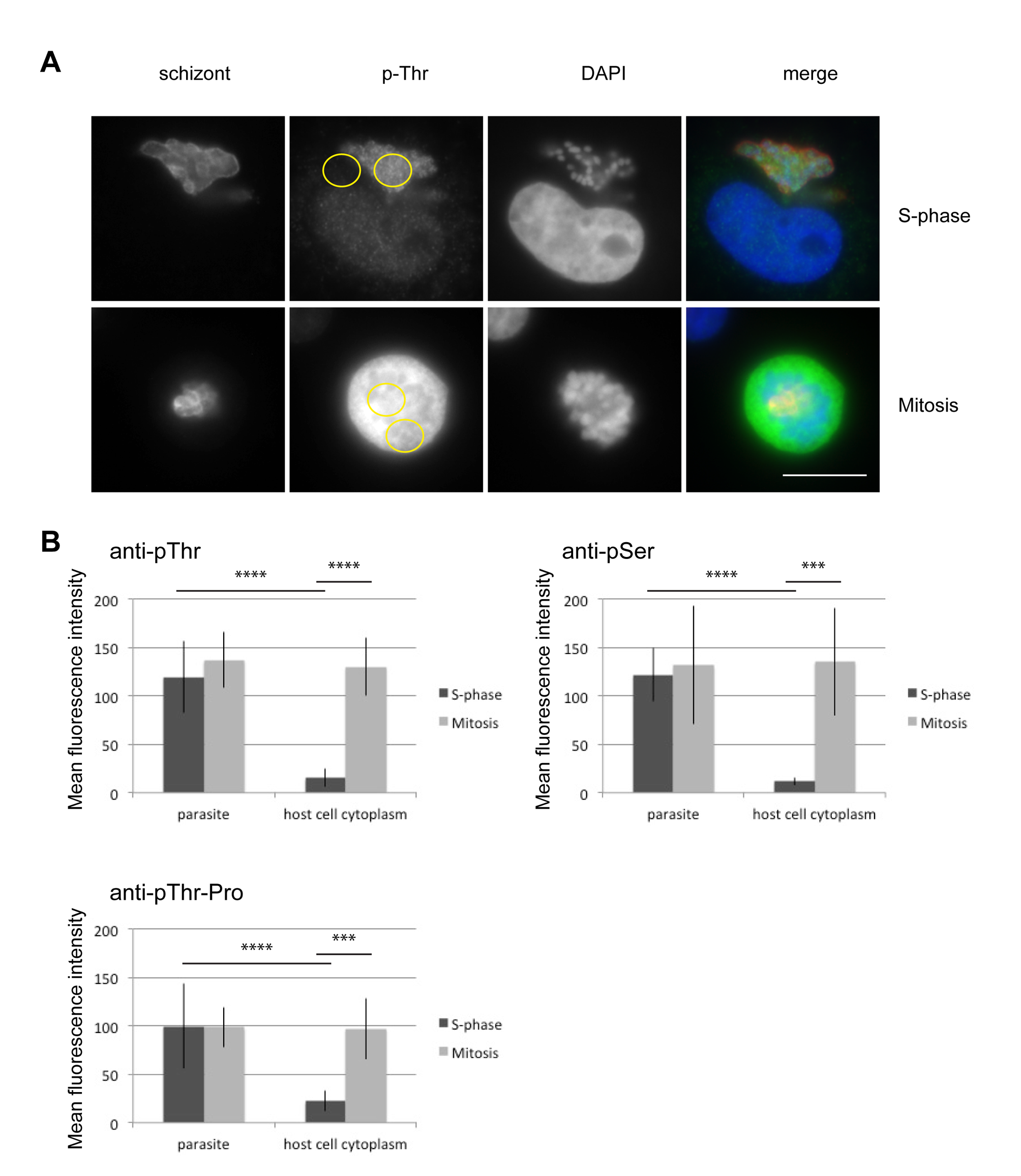

Supplement: Figure S2 — Quantified fluorescence intensity on parasite or in host cell cytoplasm. A: Immunofluorescence signal of unsynchronised TaC12 cells in S-phase or mitosis were analysed using ImageJ. Images were captured using the same exposure time for each cell. Mean fluorescence intensity was calculated in an area at the parasite or in the host cell cytoplasm (Yellow circles). A representative image following p-Thr labelling is show. B: Comparison of the mean fluorescence intensity of the phospho-epitope specific antibodies p-Thr, pThr-Pro and p-Ser at the parasite and in the host cell cytoplasm in mitosis and in S-phase. Statistically significant differences were observed between S-phase and mitosis samples for the host cell cytoplasm intensity for each antibody used (pThr p = 7×10^−8^, pThr-Pro p = 0.0014, Ser p = 0.0004), and between parasite and host cell in S-phase samples (pThr p = 1.7×10^−9^, pThr-Pro p = 2.7×10^−5^, pSer p = 3×10^−12^). **** denotes a p value <0.0001, while *** denotes a p value between 0.001 and 0.0001 (unpaired t-test, two-tailed). (TIF) [file pone.0103821.s002.tif]

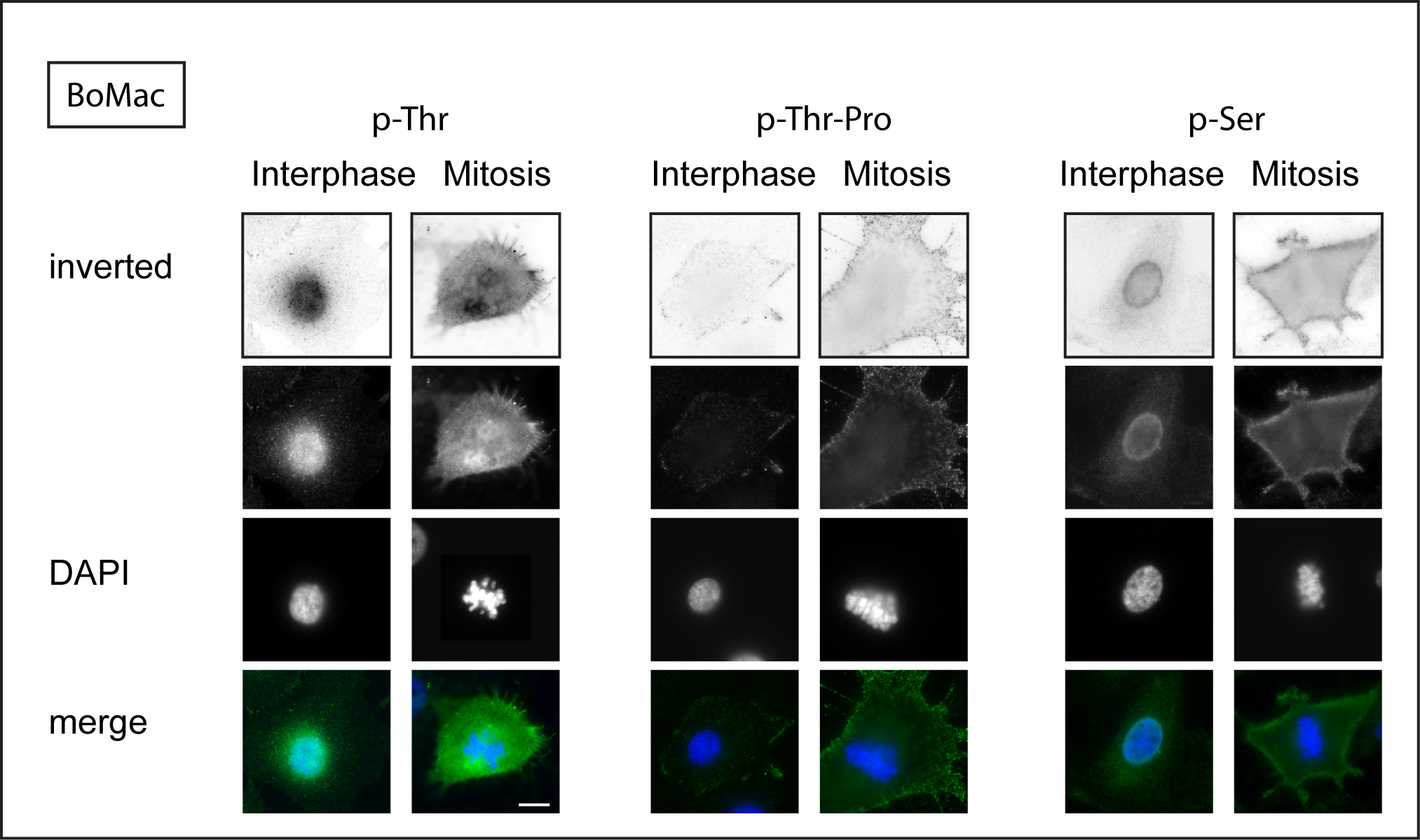

Supplement: Figure S3 — Detection of p-Thr, p-Ser and p-Thr-Pro epitopes in uninfected bovine macrophages (BoMAC) during host cell interphase and mitosis (TIF) [file pone.0103821.s003.tif]

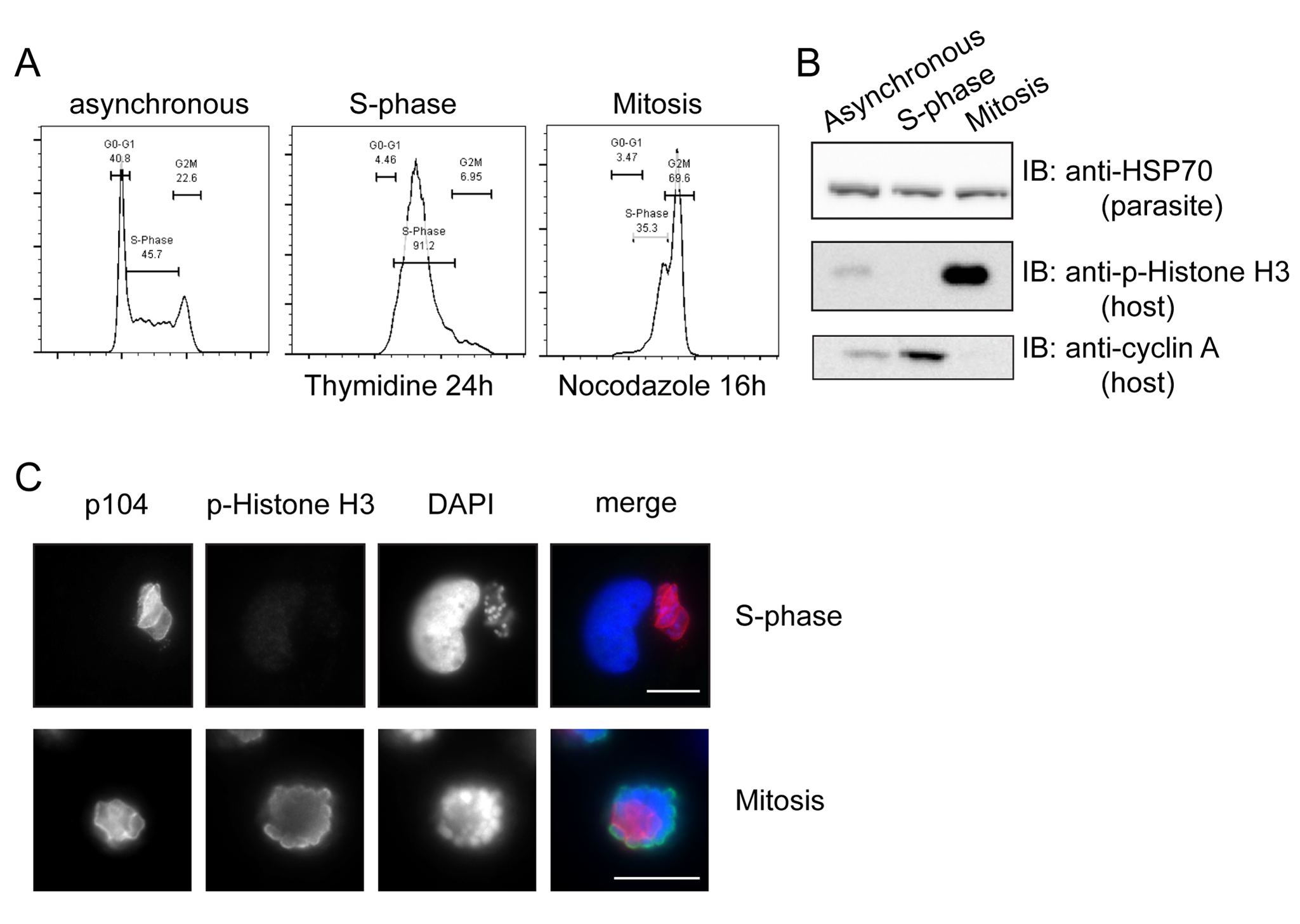

Supplement: Figure S4 — Synchronisation of TaC12 cells in S- or M-phase. A: Asynchronous TaC12 cells and cells incubated for 24 h in thymidine (S-phase) or 16 h in nocodazole (M-phase) were fixed in 80% ethanol and the DNA content was labelled with propidium iodide prior to FACS analysis. B: Lysates from TaC12 cells (unsynchronised, S-phase or M-phase) were analysed by Western blot using anti-cyclin-A and anti-p-Histone H3 antibodies. As a loading control anti-Theileria-HSP70 was used. C: Following synchronisation TaC12 cells were fixed with 4% PFA and labelled with a polyclonal anti-schizont antibody and anti-p-Histone H3. DNA was visualised with DAPI. Merge: anti-p-Histone3 (green), anti-schizont (red) and DAPI (blue). Scale bar represents 10 µm. (TIF) [file pone.0103821.s004.tif]

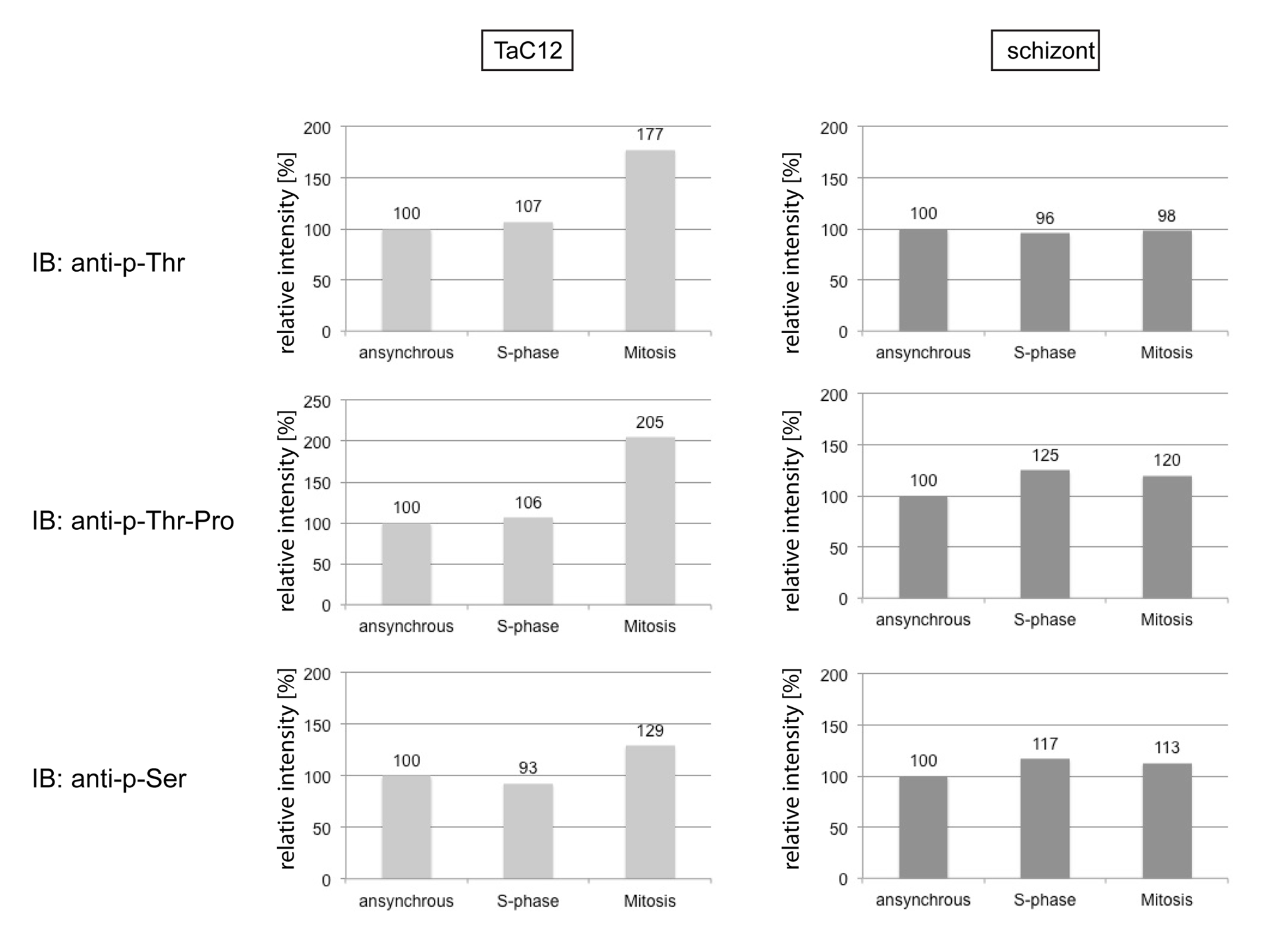

Supplement: Figure S5 — Relative signal intensity following western blotting of TaC12 and schizont lysates with anti-p-Thr, p-Thr-Pro and p-Ser antibodies. Relative intensities were measured using ImageJ, and correspond to the western blots shown in figure 5. (TIF) [file pone.0103821.s005.tif]

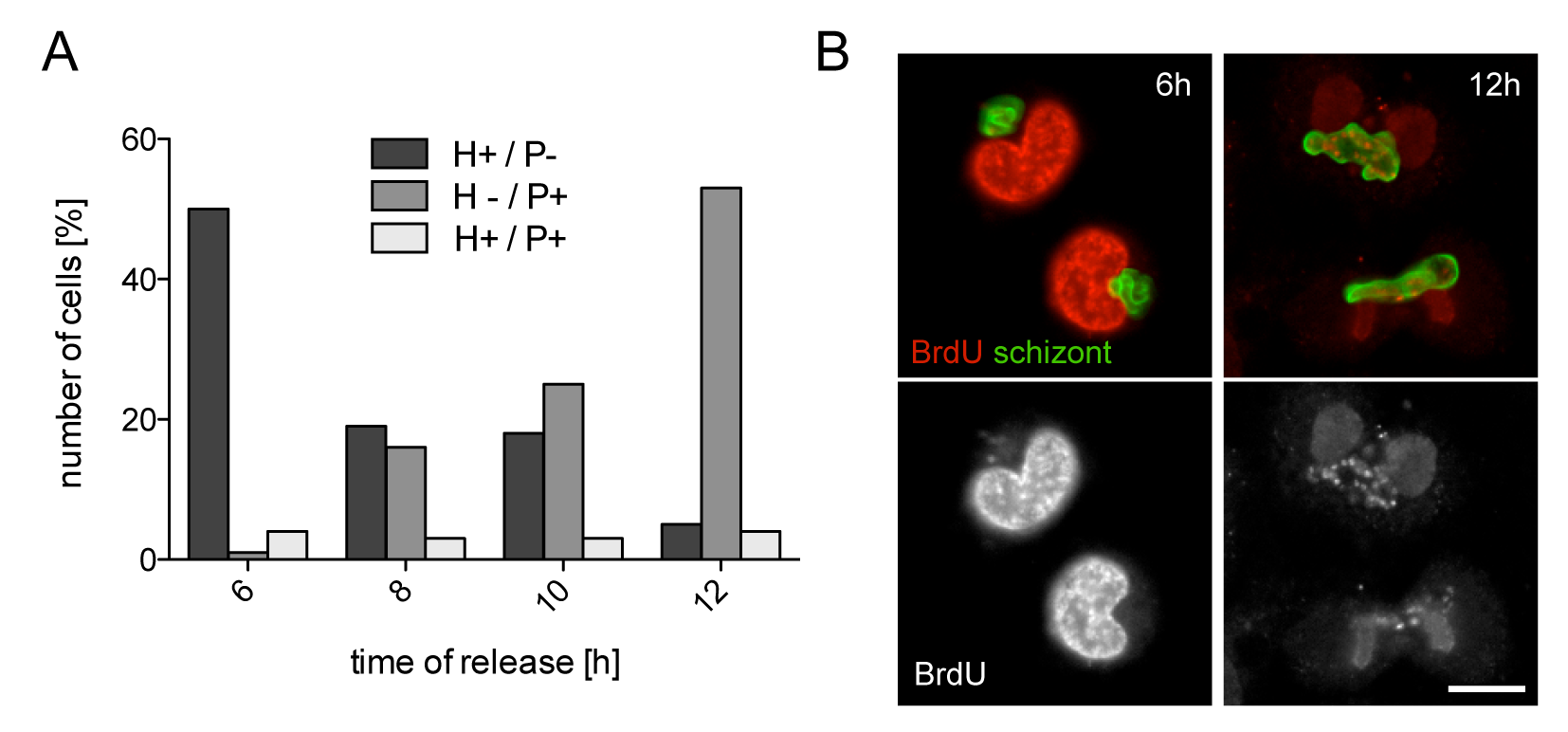

Supplement: Figure S6 — Parasite DNA replication occurs as the host cell progresses through mitosis. A: TaC12 cells were synchronised in S-phase with thymidine treatment, and released into fresh medium. BrdU (10 µM) was added to the culture 2 hours prior to analysis at 6, 8, 10 and 12 hours after thymidine release. Cells were fixed with 4% PFA and BrdU incorporation into host (H) and parasite (P) nuclei was analysed by IFA. Quantification of cells that had incorporated no BrdU (H−/P−) are excluded from the graph for clarity. n = 70−120 cells per time point. B: Representative images of cells at 6 hours (left) and 12 hours (right) post thymidine release are shown. The schizont is labelled green and BrdU is red. Scale bar represents 10 µm. (TIF) [file pone.0103821.s006.tif]
