# Supplementary material for: Cell Cycle-Dependent Phosphorylation of Theileria annulata Schizont Surface Proteins
Source: PLoS One. 2014 Jul 31;9(7):e103821. doi: 10.1371/journal.pone.0103821 (PMC4117643; doi:10.1371/journal.pone.0103821)
Supplement: Figure S7 — TaSP (TA17315) and p104 (TA08425) protein and phosphopeptide abundances. A: Three phosphorylation-sites were detected in TaSP. Two phosphorylated residues were found with a higher abundance in S-phase (p<0.01). Data were analysed with Progenesis. The max fold change for each significant (p<005) paired scanning event for peptides that were differentially detected between S- and M-phase are shown in a table (data extracted from table S6). The normalised peptide abundance for peptide SSSFSRINEDCC in S-phase and mitosis samples is presented as a histogram (consensus of all paired scanning events). B: 14 phospho-sites in p104 were detected (bold in the protein-sequence). Two detected phosphorylated peptides (corresponding to four phospho-sites) were more abundantly detected in S-phase samples (p<0.002). The max fold change for each significant (p<005) paired scanning event for peptides that were differentially detected between S- and M-phase are shown (data extracted from table S6). The normalised peptide abundance for peptides RPVSPQRPVSPR and SKSFDDLTTVR in S-phase and mitosis samples (consensus of all paired scanning events) is shown. The sequence corresponding to p104521−634 is underlined (dashed). C: Protein abundance of the Theileria surface proteins p104 and TaSP in the “Global”-analysis using Progenesis. (DOCX) [file pone.0103821.s007.docx]

A

| Amino Acid | Sequence | Modifications | Max fold change | Highest mean condition | Anova |
| --- | --- | --- | --- | --- | --- |
| Ser305 | SSSFSRINEDCC | [3] S+79.97\|[11] C+57.02\|[12] C+57.02 | 350.5196346 | S-phase | 0.008146 |
| Ser303 | SSSFSRINEDCC | [1] S+79.97\|[11] C+57.02\|[12] C+57.02 | 350.5196346 | S-phase | 0.008146 |
| Ser305 | SSSFSR | [3] S+79.97 | 55.98444705 | S-phase | 0.046755 |


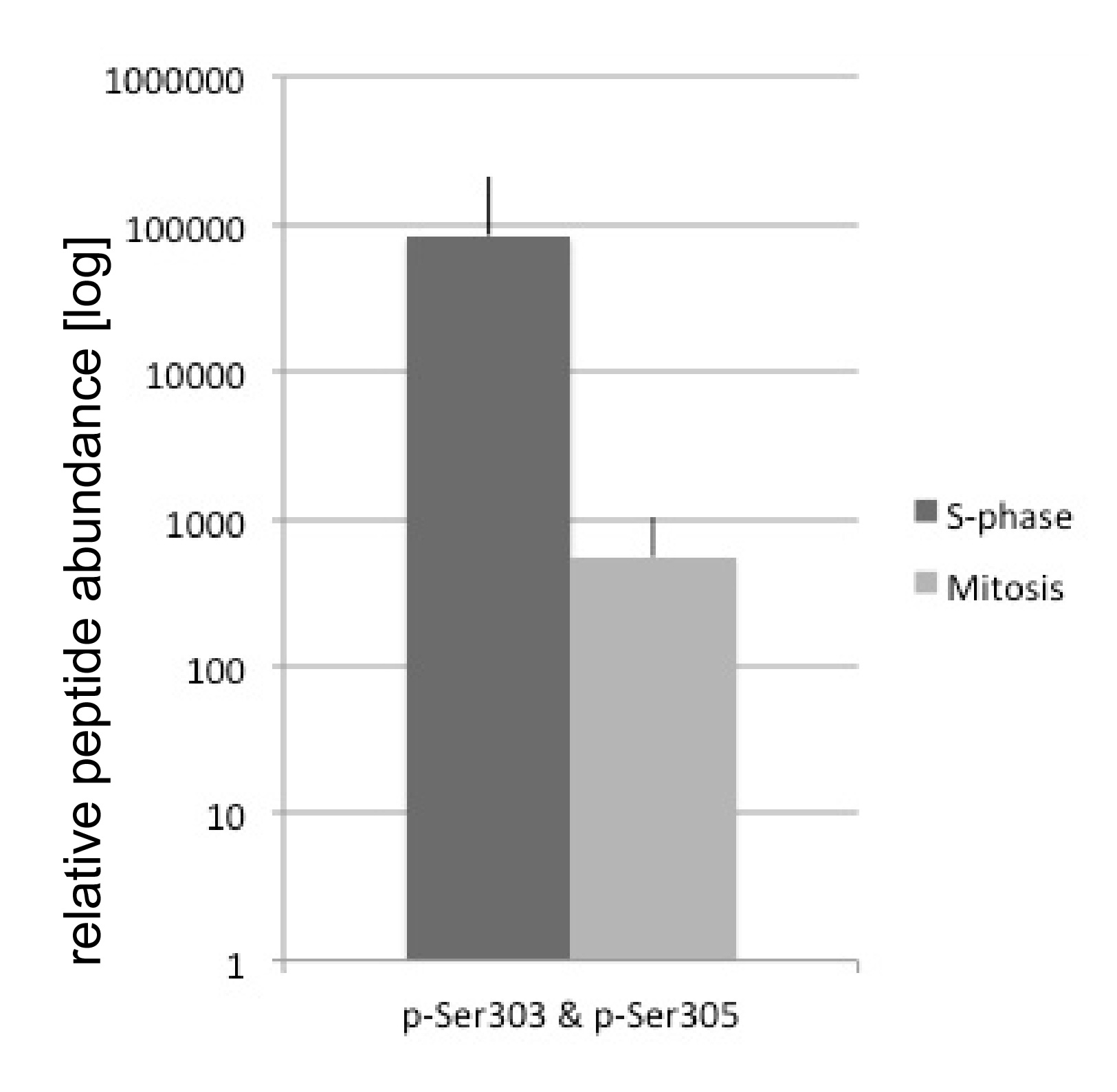


All detected phosphorylation sites in TaSP:

MKFFYLFVLFPILLKFCECGPFLPLDRQLNPIDFDPNDDQHPLDPDQLIDQIEPSEQPAQQEPIEPQQPTQPSTEPEELQPETVTVEVPEPVTSEEPKESDQTEEQKHEEPEASPAPEPVDEPAVHATESTPTKASSSGDGAAVCHGKHHDYDSDGKESKSDHDKRPKDKKPFVPKTSQCCGSYFTNSYKITVAFDWWLCDKPWQYALTLLALFGFSLLSPCLKAYREVLRAKAIRSFIFDCFLTHLFLFLIAFCAYALDFLLMLVVMTFNVGVFFAVITGYTVGYLVSSLAYSTLRSHPAR**S**S**S**FSRINEDCC

B

| Amino acid | Sequence | Modifications | Max fold change | Highest mean condition | Anova |
| --- | --- | --- | --- | --- | --- |
| Ser601, 607 | RPVSPQRPVSPR | [4] S+79.97\|  [10] S+79.97 | 9239.15 | S-phase | 0.00057 |
| Ser802 | SKSFDDLTTVR | [3] S+79.97 | 37.31 | S-phase | 0.00125 |
| Ser800 | SKSFDDLTTVR | [1] S+79.97 | 37.31 | S-phase | 0.00125 |
| Ser800 | SKSFDDLTTVREK | [1] S+79.97 | 30.63 | S-phase | 0.01095 |
| Ser802 | SFDDLTTVR | [1] S+79.97 | 10.56 | S-phase | 0.01166 |
| Ser802 | SKSFDDLTTVREK | [3] S+79.97 | 351.2 | S-phase | 0.01219 |
| Ser802 | SKSFDDLTTVR | [3] S+79.97 | 5.62 | S-phase | 0.01694 |
| Ser800 | SKSFDDLTTVR | [1] S+79.97 | 5.62 | S-phase | 0.01694 |
| Ser802 | SKSFDDLTTVR | [3] S+79.97 | 27.84 | mitosis | 0.02995 |


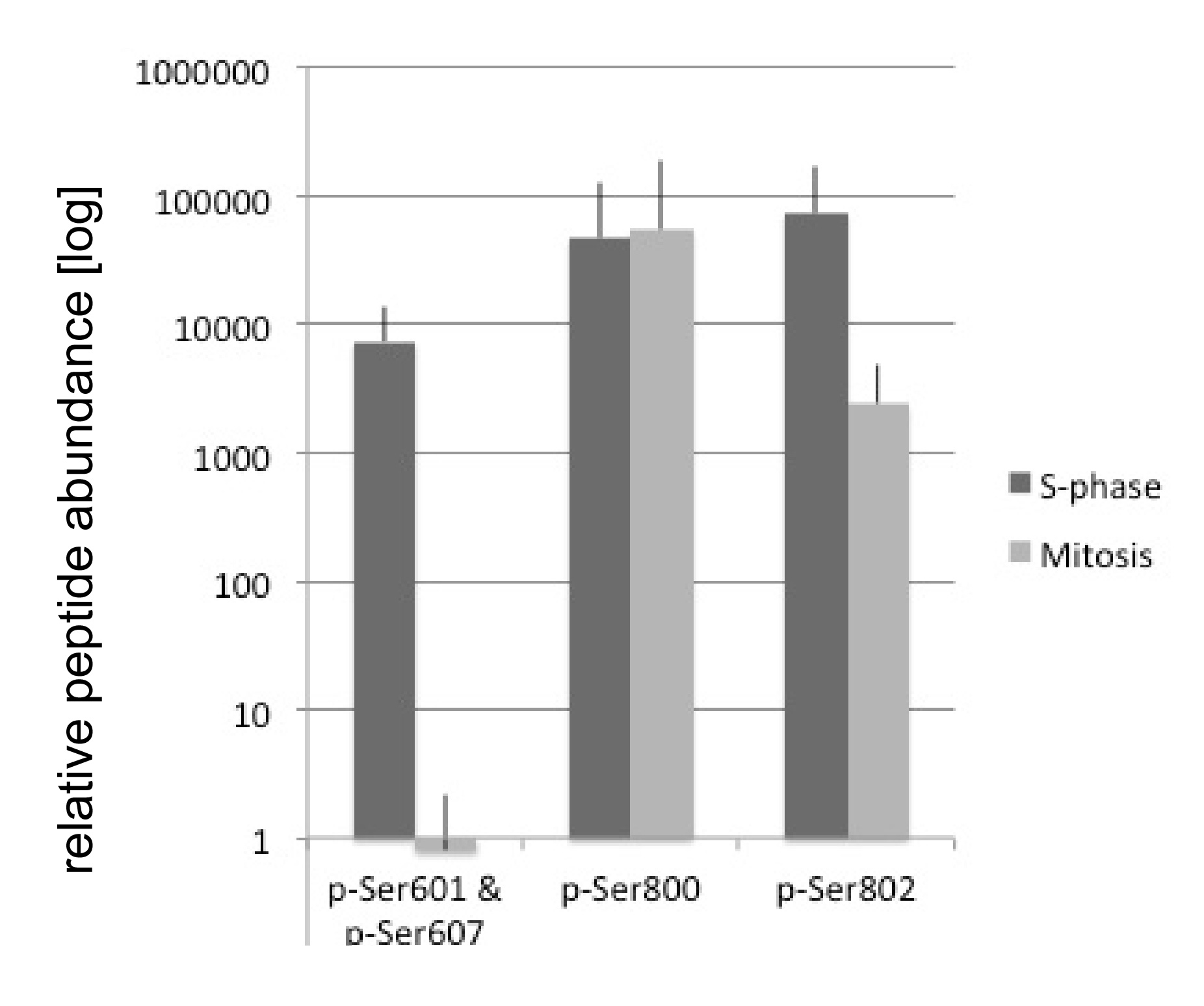


All detected phosphorylation sites in p104:

MKFLVLLFNILCLFPILGADELVM**S**PIPTTDVQPKVTFDINSEVSSGPLYLNPVEMAGVK

YLQLQRQPGVQVHKVVEGDAVIWENEEMPLYTCAIVTQNEAPYMAYVELLEDPDLIFFLK

EGDQWAPIPEDQYLARLHQLRQAIHTESFFSLNLSFQHENYKYEMVSSFQHSVKMVVFTP

KNGHICKMVYDKNVRIFKAFYNEYVTSVIGFFRGLKLLLLNIFVIDDHGMVGNKYFQLLE

DKYAPISVQGYVATIPKLKDFAEPYHPIILDISDIDYVNFYLGDATYHDPGFKIVPKTPQ

CITKVVDGNEVIYESSNPSVECVYKVTYYDKKNEPMLRLDLNHSPPSYTQYFAKRDGVWV

TSTYIDLEEKIEELQDHRSTELDVMFMSDKDLNVVPLTNGNLEYFMVTPKPHRDIIIVFD

GSEVLWYYEGLENHLVCTWIYVTEGAPRLVHLRVKDRIPQNTDIYMVKFGEYWVRISKTQ

YTQEIKKLIKKSKKKLPSIEEEDSDKHGGPPKGPEPPKGPGHSSSESKEHEDAKESKEPK

EHGSPKETKEGEIAKKPGSAKEHRPSKIPVFTKKSEITKKSKSPKRPESPKRPESPKRPV

**S**PQRPV**S**PRRPESPKLPKSPK**S**PKRPESLDIPKSPKRPESPKSPKSPKSPK**S**PKVPFDPK

FKEKLYDSYLDKAAKTKEAVTLPPVLPTDESFTHTPIGEPTAEQPDDIEPIEESVFIKET

GILTEEVKTEDIHSETGEPEVPKRPDSPTKHSPKPTGTHPSLPKKRRR**S**DGLAL**STT**DLE

SEAGRTLRDPTGKIVTMKR**S**K**S**FDDL**T**TVREKEHMGAEIRKIVVDDDG**T**EADDEDTHP**S**K

EKHLSTVRRRRPRPKKSSKSSKPRKPDSAFVPSLLFIFLVSLIVGIL

C

| Source | Accession | Peptide count | Anova (p) | Max fold change | Highest mean condition |
| --- | --- | --- | --- | --- | --- |
| Progenesis_Global | TA08425 | 15 | 0.02 | 1.19 | S-Phase |
| Progenesis_Global | TA17315 | 2 | 0.29 | 1.46 | Mitosis |
